# CCT3/ACTN4/TFRC axis protects hepatocellular carcinoma cells from ferroptosis by inhibiting iron endocytosis

**DOI:** 10.1186/s13046-024-03169-7

**Published:** 2024-08-29

**Authors:** Huihui Zhu, Qiuhong Liu, Qinna Meng, Lingjian Zhang, Siwei Ju, Jiaheng Lang, Danhua Zhu, Yongxia Chen, Nadire Aishan, Xiaoxi Ouyang, Sainan Zhang, Lidan Jin, Lanlan Xiao, Linbo Wang, Lanjuan Li, Feiyang Ji

**Affiliations:** 1 Zhejiang Provincial Key Laboratory of Pancreatic Disease, The First Affiliated Hospital, Zhejiang University School of Medicine, Hangzhou, 310000 Zhejiang China; 2 Department of Infectious Diseases, Affiliated Hangzhou First People’s Hospital, Westlake University School of Medicine, Hangzhou, 310000 Zhejiang China; 3 State Key Laboratory for Diagnosis and Treatment of Infectious Diseases, The First Affiliated Hospital, Zhejiang University School of Medicine, Hangzhou, 310000 Zhejiang China; 4 School of Basic Medical Sciences and Forensic Medicine, Hangzhou Medical College, Hangzhou, 310000 Zhejiang China; 5 Department of Surgical Oncology, Sir Run Run Shaw Hospital, Zhejiang University School of Medicine, Hangzhou, 310000 Zhejiang China; 6 Provincial Clinical Research Center for CANCER, Hangzhou, 310000 Zhejiang China; 7 Shulan (Hangzhou) Hospital Affiliated to Zhejiang Shuren University Shulan International Medical College, Hangzhou, 310000 Zhejiang China; 8 Department of Rheumatology, The First Affiliated Hospital, Zhejiang University School of Medicine, Hangzhou, 310000 Zhejiang China

**Keywords:** Endosome, Cell death, Proteomics, Drug resistance, Cancer

## Abstract

Sorafenib is widely used in treating advanced hepatocellular carcinoma (HCC). However, its effectiveness in prolonging patient survival is limited by the development of drug resistance. To systematically investigate the resistance mechanisms of Sorafenib, an integrative analysis combining posttranslational modification (PTM) omics and CRISPR/Cas9 knockout library screening was conducted. This analysis identified ubiquitination at lysine 21 (K21) on chaperonin-containing TCP1 subunit 3 (CCT3) as being associated with Sorafenib resistance. Transcriptomic data from HCC patients treated with Sorafenib revealed that CCT3 expression was lower in responders compared to non-responders. Experimentally, inhibiting the expression of CCT3 sensitized HCC cells to Sorafenib and enhanced Sorafenib-induced ferroptosis. Additionally, CCT3 was found to interact with ACTN4, hindering the recycling of transferrin receptor protein 1 (TFRC) to the cell membrane, thus obstructing iron endocytosis. Mechanistically, the inhibition of ferroptosis by CCT3 depends on the deubiquitination of K6-linked non-degradative ubiquitination at its K21, which occurs upon Sorafenib treatment. Moreover, CCT3 knockdown enhanced the anti-tumor effects of Sorafenib in nude mice. In summary, we have identified a novel function of the chaperone protein. Targeting the CCT3/ACTN4/TFRC axis offers a promising strategy to enhance ferroptosis and overcome Sorafenib resistance in HCC.

**Supplementary Information:**

The online version contains supplementary material available at 10.1186/s13046-024-03169-7.

## Introduction

Liver cancer ranks sixth globally among all cancer types and is the third leading cause of cancer-related deaths [1]. Among primary liver cancers, hepatocellular carcinoma (HCC) is the most prevalent. In the initial stages of HCC, surgical intervention remains the prevailing treatment modality. However, approximately 50% of advanced HCC cases necessitate Sorafenib and local ablation as the primary therapeutic approach [2, 3]. Sorafenib, an orally administered multi-target tyrosine kinase inhibitor, effectively impedes the proliferation and angiogenesis of tumor cells by targeting vascular endothelial growth factor receptor (VEGFR), platelet-derived growth factor receptor (PDGFR), Raf family kinases, among others. Consequently, the survival rates of HCC patients experience some improvement [4, 5]. Despite its effectiveness in treating HCC, Sorafenib’s impact on patients’ median overall survival is limited, extending it by merely three months [4]. The emergence of drug resistance poses a significant challenge to the efficacy of Sorafenib treatment in HCC patients. Enhancing our comprehension of the molecular mechanisms underlying Sorafenib resistance in HCC holds the potential to enhance disease prognosis and facilitate the identification of therapeutic targets capable of circumventing chemoresistance.

Epigenetics, transport processes, tumor microenvironment, and regulated cell death have been identified as potential triggers and contributors to the development of Sorafenib resistance [6]. In the realm of therapy resistance mechanisms, there has been a growing focus on ferroptosis, which exhibits unique characteristics in terms of morphology, biochemistry, and genetics compared to apoptosis, autophagy, necrosis, and other forms of cell death [7]. In the process of ferroptosis, there is a significant accumulation of iron and lipid peroxidation. The regulatory mechanisms primarily involve Xc- system, glutathione peroxidase 4(GPX4) activity, and reactive oxygen species (ROS) production [8]. Sorafenib, unlike other tyrosine kinase inhibitors, has the ability to induce ferroptosis in various cancer cell lines [9]. Research has indicated that ferroptosis is linked to Sorafenib resistance in HCC. For instance, the depletion of intracellular iron stores using deferoxamine (DFO) has been shown to protect HCC cells from the cytotoxic effects of Sorafenib [10]. However, further investigation is required to elucidate the molecular mechanism of ferroptosis in Sorafenib resistance.

CCT3, a subunit of the chaperonin containing TCP1 (CCT) complex, is a constituent of the TCP1 ring complex (TRiC) family, comprising eight subunits (CCT1-CCT8). The CCT complex serves as a molecular chaperone essential for the proper folding of actin and tubulin, key components of cytoskeletal microfilaments and microtubules. Additionally, the individual subunits of the CCT complex have been observed to possess independent functionalities [11]. Increased expression of CCT3 has been associated with unfavorable prognoses in patients with HCC, while its depletion has been shown to induce apoptosis and impede the proliferation of liver cancer cells [12, 13]. Furthermore, existing research has demonstrated that CCT3 exerts inhibitory effects on apoptosis, while simultaneously promoting cell proliferation and metastasis in various malignant tumors, including breast cancer, melanoma, and lung cancer [14–16]. However, the potential contribution of CCT3 to Sorafenib resistance in HCC remains uncertain, although it has been observed to induce cisplatin resistance in lung adenocarcinomas through its targeting of JAK2/STAT3 [17]. Moreover, the inhibition of ferroptosis by CCT3 has been implicated in the development of lung cancer, while its presence can lead to the inhibition of lipid metabolism and the promotion of lipid accumulation in liver cancer [18, 19].

Sorafenib has the potential to induce signal transduction and posttranslational modification (PTM) alterations in hepatoma cells. Consequently, the identification of proteins exhibiting significant PTM changes can serve as a means to screen for molecules associated with Sorafenib resistance. Mass spectrometry-based protein PTM omics enables the simultaneous identification and quantification of numerous protein PTMs, offering advantages such as high throughput, precision, and the ability to directly localize modifications to specific amino acid residues. As a result, this methodology has gained increasing prominence in cancer research in recent years. Wang et al. have identified phosphorylated PTPN11 and PLCG1 as potential mediators of oncogenic pathway activation in human glioblastoma [20]. Similarly, another study utilizing this approach has demonstrated the critical role of SYVN1 in the progression of HCC metastasis [21]. Genome-wide CRISPR/Cas9 screening involves the utilization of CRISPR/Cas9 technology to construct a comprehensive gene mutation library of a particular species. This library is then subjected to functional screening, PCR amplification, and deep sequencing to identify genes associated with a specific phenotype. Lai Wei and colleagues employed this approach to conduct a screening of 986 genes implicated in Sorafenib resistance in 97 L cells [22]. Huang and colleagues discovered that the absence of DUSP4 leads to the development of lenvatinib resistance in HCC [23]. The integration of PTM omics and genome-wide Cas9 screening enables a comprehensive identification of proteins associated with specific biological processes, encompassing signaling pathways and phenotypes. This methodology significantly enhances the precision of screening and provides valuable insights for subsequent mechanistic investigations.

## Results

### Integrating PTM omics and CRISPR/Cas9 knockout screening identified CCT3 as a resistance gene to Sorafenib

**Fig. 1:**
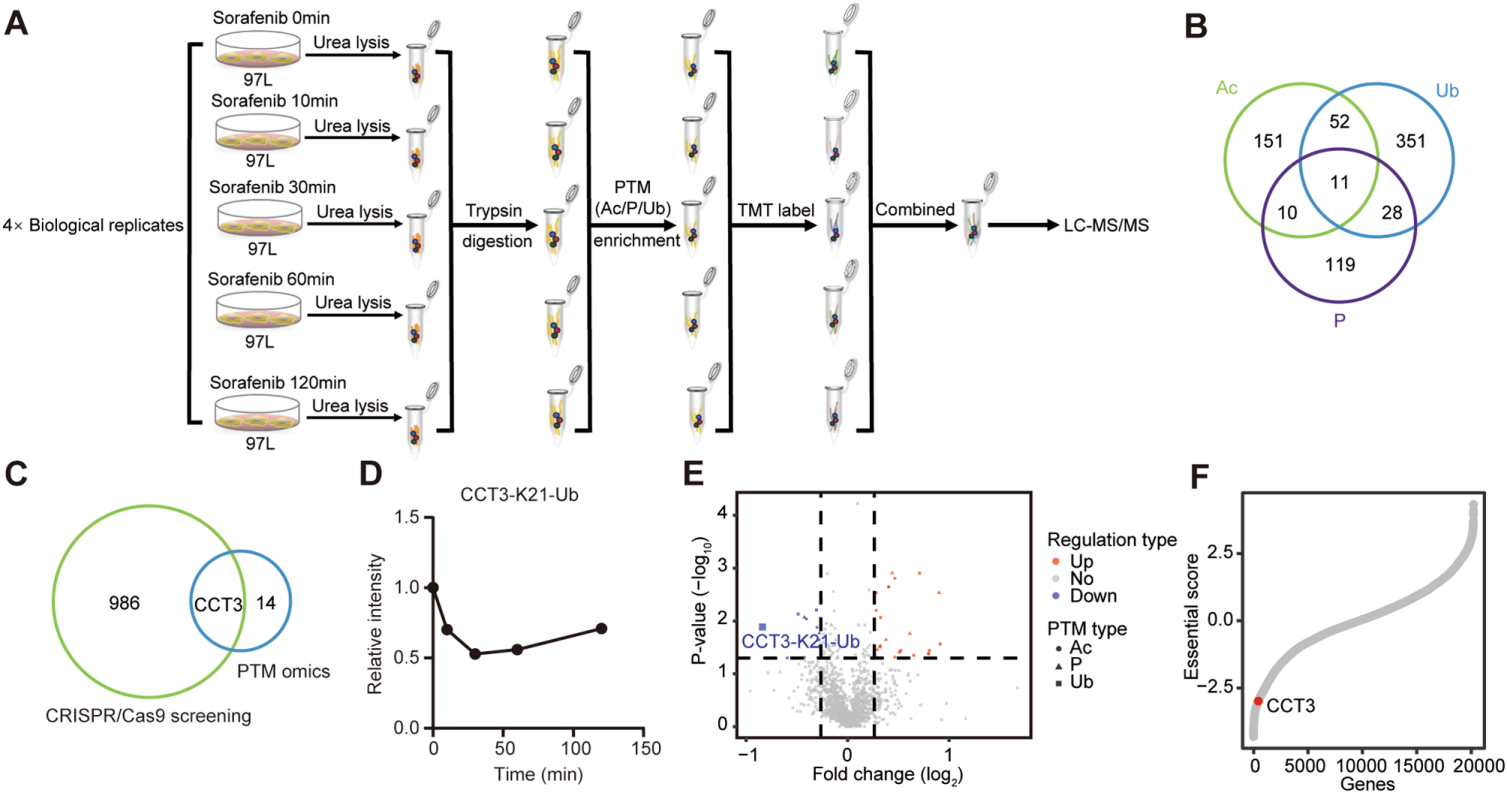
Combined PTM omics and genome-wide CRISPR/Cas9 knockout identified CCT3 as an important gene for Sorafenib sensitivity. **A** Flow diagram depicting the posttranslational modification (PTM) omics of acetylation (Ac), phosphorylation (P), and ubiquitination (Ub) in 97 L cells treated with Sorafenib was presented. **B** Venn diagram showing the overlap of identified proteins between three posttranslational modifications. **C** Venn diagram displaying the overlap of proteins across PTM omics screening and genome-wide CRISPR/Cas9 knockout screening. **D** The line chart shows the change of the ubiquitination of CCT3 at lysine 21 (CCT3-K21-Ub) in PTM omics after Sorafenib treatment. **E** Volcano plot showing the regulated PTM sites in 97 L cells treated with Sorafenib for 1 h. **F** The essential score of CCT3 targeting sgRNAs (red dot) in genome-wide CRISPR/Cas9 knockout screening. Negative essential scores represent negative selection, and positive essential scores represent positive selection

In this study, we conducted PTM omics analysis to quantify alterations in protein phosphorylation, acetylation, and ubiquitination following Sorafenib treatment in HCC cells (Fig. 1A, Supplementary Table 1). The genome-wide CRISPR/Cas9 knockout library screening was previously carried out by Lai et al. [22]. The experimental parameters for the selection of 97 L cells and the administration of 7 μm Sorafenib in PTM omics were adopted from the CRISPR/Cas9 knockout library screening to ensure the congruity of the findings between the two approaches. Additionally, we found that 97 L exhibits higher tolerance to sorafenib compared to other liver cancer cell lines (Fig. S1A). Using PTM omics, we successfully detected a total of 224 acetylated modified proteins, 168 phosphorylated modified proteins, and 442 ubiquitinated modified proteins in 97 L cells. Interestingly, 11 proteins exhibited all three types of PTMs (Fig. 1B). Upon treatment with Sorafenib, we observed alterations in the PTMs of certain proteins, primarily an increase in their abundance (Fig. S1B). Furthermore, our analysis of gene ontology (GO) terms enrichment indicated that these proteins were associated with membrane raft, chaperonin-containing T-complex, and receptor tyrosine kinase binding (Fig. S1C-D). Among the list of 14 proteins whose PTM was significantly altered following treatment with Sorafenib, it was found that only CCT3 had an impact on Sorafenib resistance as determined through screening with a genome-wide CRISPR/Cas9 knockout library (Fig. 1C). Specifically, the ubiquitination of lysine residue 21 (K21) in CCT3 was notably reduced after Sorafenib treatment, particularly at the 30-minute time point, indicating the crucial involvement of CCT3 in the signaling pathway of 97 L cells in response to Sorafenib (Figs. 1D-E, S1E). Furthermore, the presence of CCT3 targeting single guide RNA (sgRNA) was noticeably diminished in Sorafenib-treated 97 L cells, suggesting that the depletion of CCT3 may enhance sensitivity to Sorafenib (Fig. 1F). The findings from the aforementioned methodologies indicate that CCT3 exhibits responsiveness to Sorafenib stimuli and can effectively inhibit its activity. Additionally, Sorafenib disrupts global phosphorylation, acetylation, and ubiquitination, suggesting their involvement in cellular responses (Fig S1F). The iceLogo analysis further revealed conserved patterns of peptides with notable PTM alterations (Fig. S1G).

### CCT3 promotes Sorafenib resistance in HCC cells

**Fig. 2:**
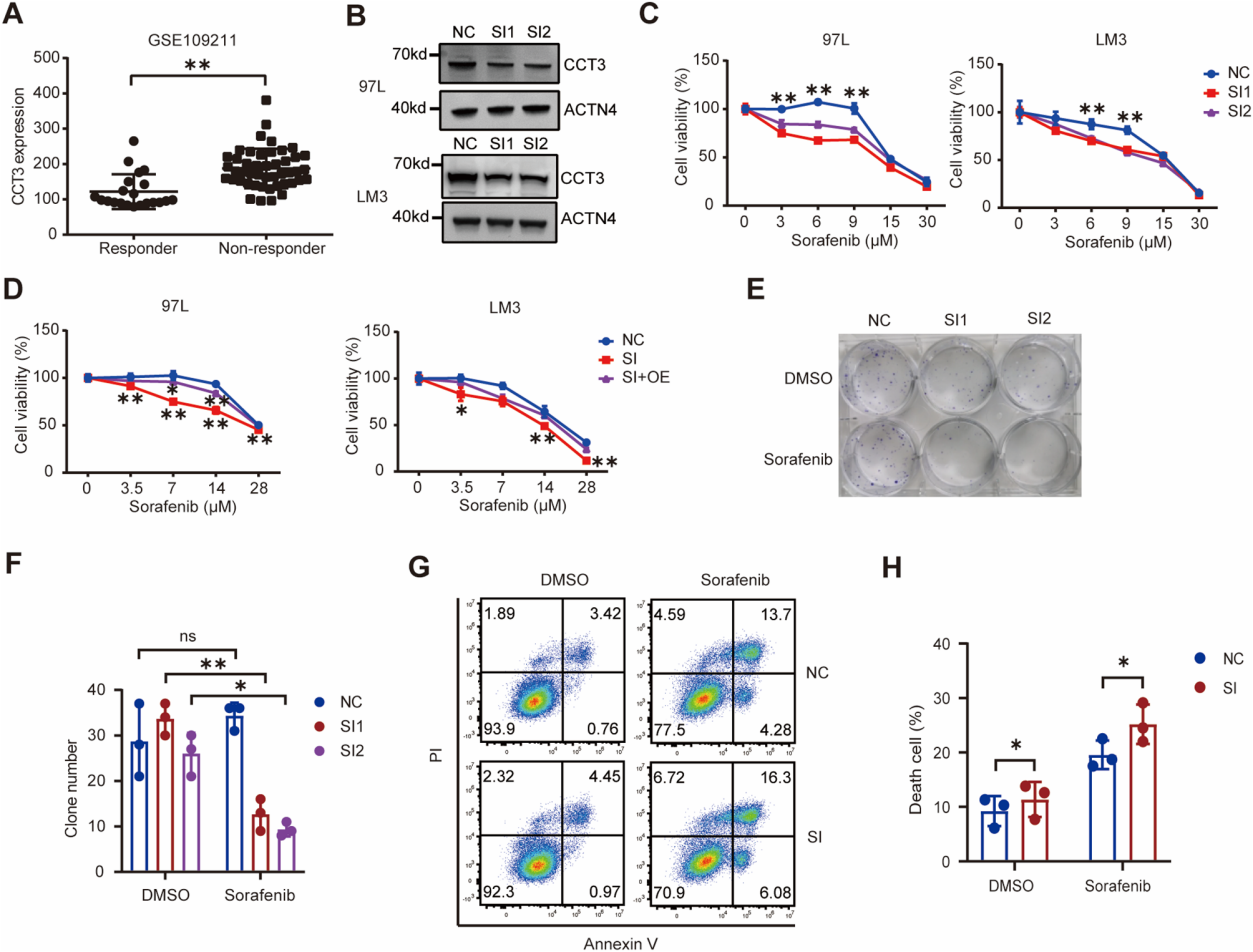
Inhibiting CCT3 sensitized HCC cells to Sorafenib. **A** The expression level of CCT3 in Sorafenib response or non-response hepatocellular carcinoma from GEO database. **B** Western blot analysis of the indicated proteins in cells treated with negative control siRNA (NC) and two CCT3 siRNA (SI1, SI2). **C** Analysis of cell viability in control and CCT3-knockdown 97 L and LM3 cells following treatment with Sorafenib for 48 h. **D** Cell viability of control, CCT3-knockdown (SI), and CCT3-overexpression (OE) after CCT3-knockdown cells following treatment with Sorafenib for 24 h. **E** Clone formation capacity of control and CCT3-knockdown 97 L cells following treatment with DMSO or 2 μM Sorafenib. **F** Statistics histogram for clone assays from three biological replicates. **G** Apoptosis of indicted 97 L cells treated with DMSO or 7 μM Sorafenib for 24 h were performed using Annexin V/PI staining. **H** Histogram showing the statistical results of apoptosis from three biological replicates. Each cell viability data is representative of three biological replicates

Through analysis of the GSE144269 transcriptomic dataset, we found that CCT3 expression is significantly higher in hepatocellular carcinoma tissue compared to adjacent non-cancerous tissue, consistent with previous research findings (Fig. S2A) [24]. In order to validate the findings obtained through the combination of two screening methods, the transcriptomic dataset GSE109211 of HCC patients treated with Sorafenib was obtained from the Gene Expression Omnibus database [25]. The analysis revealed a negative correlation between the expression level of CCT3 in HCC and the efficacy of Sorafenib (Fig. 2A). Additionally, to further investigate the impact of CCT3 on the susceptibility of HCC to Sorafenib, two small interfering RNA (siRNA) sequences targeting CCT3 were designed and utilized to suppress CCT3 expression in two HCC cell lines, namely 97 L and LM3. The knockdown efficiency of two CCT3 siRNA molecules was further confirmed through western blotting analysis in both 97 L and LM3 cells (Fig. 2B, S2B). The knockdown of CCT3 resulted in increased sensitivity of 97 L and LM3 cells to Sorafenib-induced growth inhibition (Fig. 2C). Notably, the rescue experiment demonstrated that the overexpression of CCT3 mRNA reversed the effect of CCT3 siRNA on Sorafenib sensitivity (Fig. 2D). Additionally, the knockdown of CCT3 significantly impaired the ability of 97 L and LM3 cells to form clones (Figs. 2E-F, S2C-D). Using flow cytometry, we observed a statistically significant increase in cell death following CCT3 knockdown in Sorafenib-treated 97 L cells (Fig. 2G-H). Additionally, similar results were obtained in LM3 cells, but not as prominently as in 97 L cells, which may be due to the different concentrations of SORA used (Fig. S2E-F). These results suggest that inhibiting CCT3 enhances the susceptibility of HCC cells to Sorafenib therapy.

### CCT3 promotes Sorafenib resistance by inhibiting ferroptosis

**Fig. 3:**
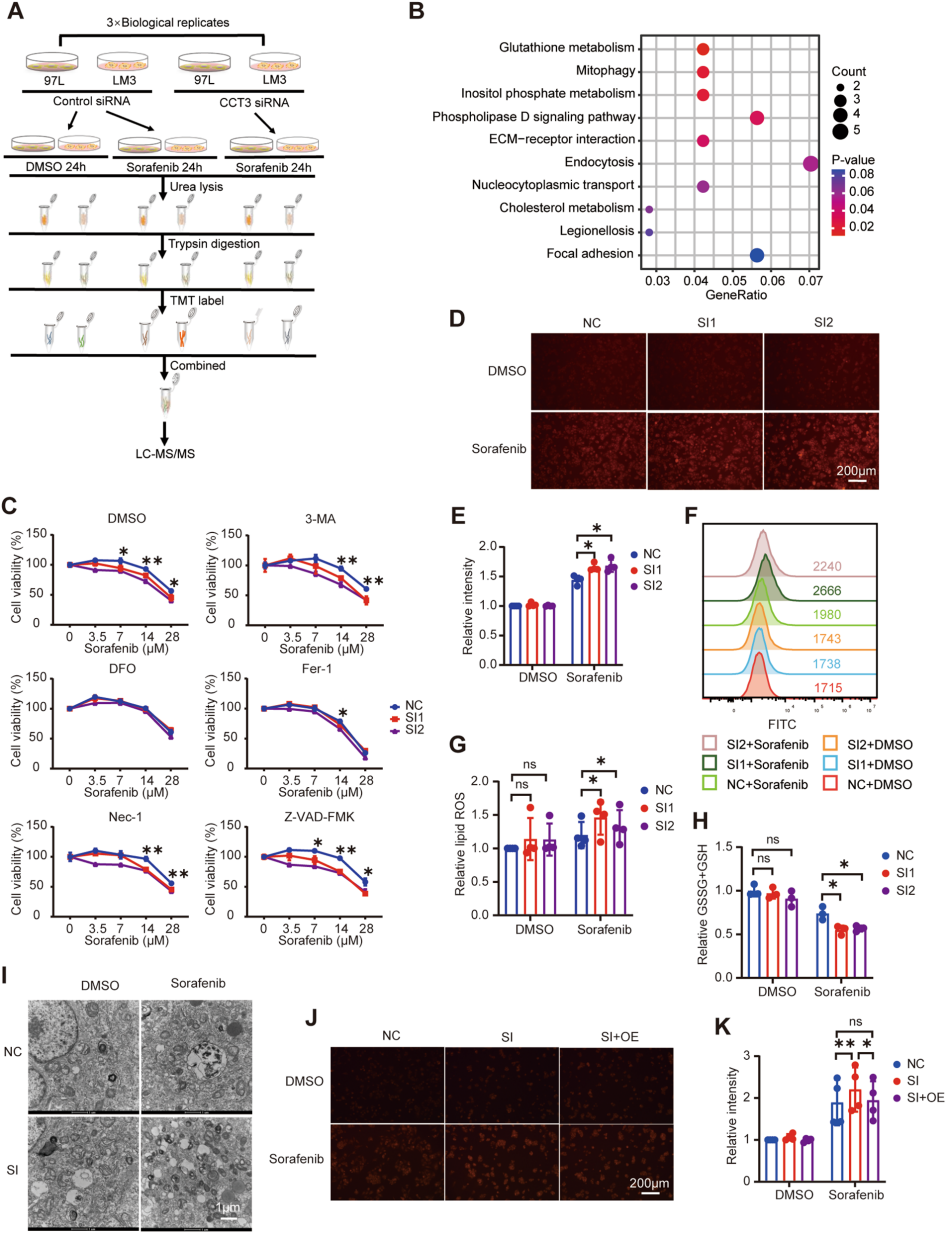
Inhibition of CCT3 promotes ferroptosis in Sorafenib-treated HCC cells. **A** Flow chart of the whole cell proteomic for 97 L and LM3 cells treated with Sorafenib and CCT3 siRNA. **B** KEGG pathway enrichment analysis of differentially expressed protein between control and CCT3-knockdown 97 L cells. **C** Cell viability of control and CCT3-knockdown 97 L cells treated with Sorafenib and indicated cell death inhibitors for 24 h. **D-E** The accumulation of iron in control and CCT3-knockdown 97 L cells were detected using FerroOrange after treated with DMSO or Sorafenib (14 μM) for 12 h. Histogram showing the statistical results of iron accumulation from four biological replicates. **F-G** 97 L cells were treated with DMSO or Sorafenib (14 μM) for 24 h, and then lipid hydroperoxides were measured. Statistics for the median Fluorescence intensity of oxidized BODIPY dyes were carried out on four biological replicates. **H** 97 L cells were treated with Sorafenib at 14 μM for 12 h, and the intracellular glutathione (GSH) level were assayed. **I** Transmission electron microscopy was used to examine the morphology of indicated 97 L cells after 24 h of treatment with Sorafenib (14 μM). **J-K** Indicated 97 L cells were treated with Sorafenib (14 μM) for 12 h, and then iron accumulation were measured and statistical analysis

To investigate the underlying mechanism through which CCT3 facilitates resistance to Sorafenib in HCC, a comprehensive analysis of cellular proteomics was conducted to identify alterations in protein expression in 97 L and LM3 cells following treatment with Sorafenib and inhibition of CCT3 expression (Fig. 3A, Supplementary Table 2). The results of our study demonstrated significant changes in protein expression in 97 L and LM3 cells upon Sorafenib treatment and CCT3 inhibition (Fig. S3A). The Kyoto Encyclopedia of Genes and Genomes (KEGG) pathway analysis indicated that the proteins that exhibited differential expression after inhibiting CCT3 were implicated in various cellular processes, including glutathione metabolism, mitophagy, and endocytosis, which are associated with intracellular iron transport and ferroptosis (Fig. 3B). The effectiveness of CCT3 knockdown was also confirmed through GO analysis, which revealed enrichment of the chaperonin-containing T-complex pathway (Fig. S3B). The analysis conducted using KEGG also revealed the involvement of ferroptosis, reactive oxygen species, and oxidative phosphorylation in the response to Sorafenib (Fig. S3C). The collective findings from the proteomics study suggest that the inhibition of CCT3 enhances Sorafenib sensitivity, potentially through the induction of ferroptosis. To confirm this hypothesis, we employed 3-Methyladenine (3-MA), Necrostatin-1 (Nec-1), Z-VAD-FMK, Deferoxamine (DFO), and Ferrostatin-1 (Fer-1) to inhibit autophagy, necrosis, apoptosis, and ferroptosis, respectively. Our results demonstrated that only the ferroptosis inhibitors DFO and Fer-1 weakened the impact of CCT3 on Sorafenib sensitivity, while the other inhibitors of cell death pathways did not (Fig. 3C). In order to investigate the central events of ferroptosis, namely iron accumulation and lipid peroxidation, we conducted further analysis. Utilizing FerroOrange, a probe for detecting ferro-ions, we observed that the downregulation of CCT3 resulted in enhanced ferrous ion accumulation in Sorafenib-treated 97 L and LM3 cells (Figs. 3D-E, S3D-E). Additionally, the knockdown of CCT3 promoted lipid peroxidation in these cells (Fig. 3F-G, S3F-G). Consistent with expectations, our findings demonstrated that the reduction of CCT3 levels led to a decrease in intracellular glutathione levels (Figs. 3H, S3H). The findings from transmission electron microscopy demonstrated that the reduction of CCT3 expression resulted in a decrease in mitochondrial volume and an increase in membrane density in 97 L and LM3 cells (Figs. 3I, S3I). Notably, the rescue experiment revealed that the overexpression of CCT3 mitigated the impact of CCT3 expression interference on the accumulation of ferrous ions in 97 L and LM3 cells treated with Sorafenib (Figs. 3J-K, S3J-K). In conclusion, CCT3 functions as an inhibitor of Sorafenib-induced ferroptosis in HCC cells.

### CCT3 interacts with ACTN4

**Fig. 4:**
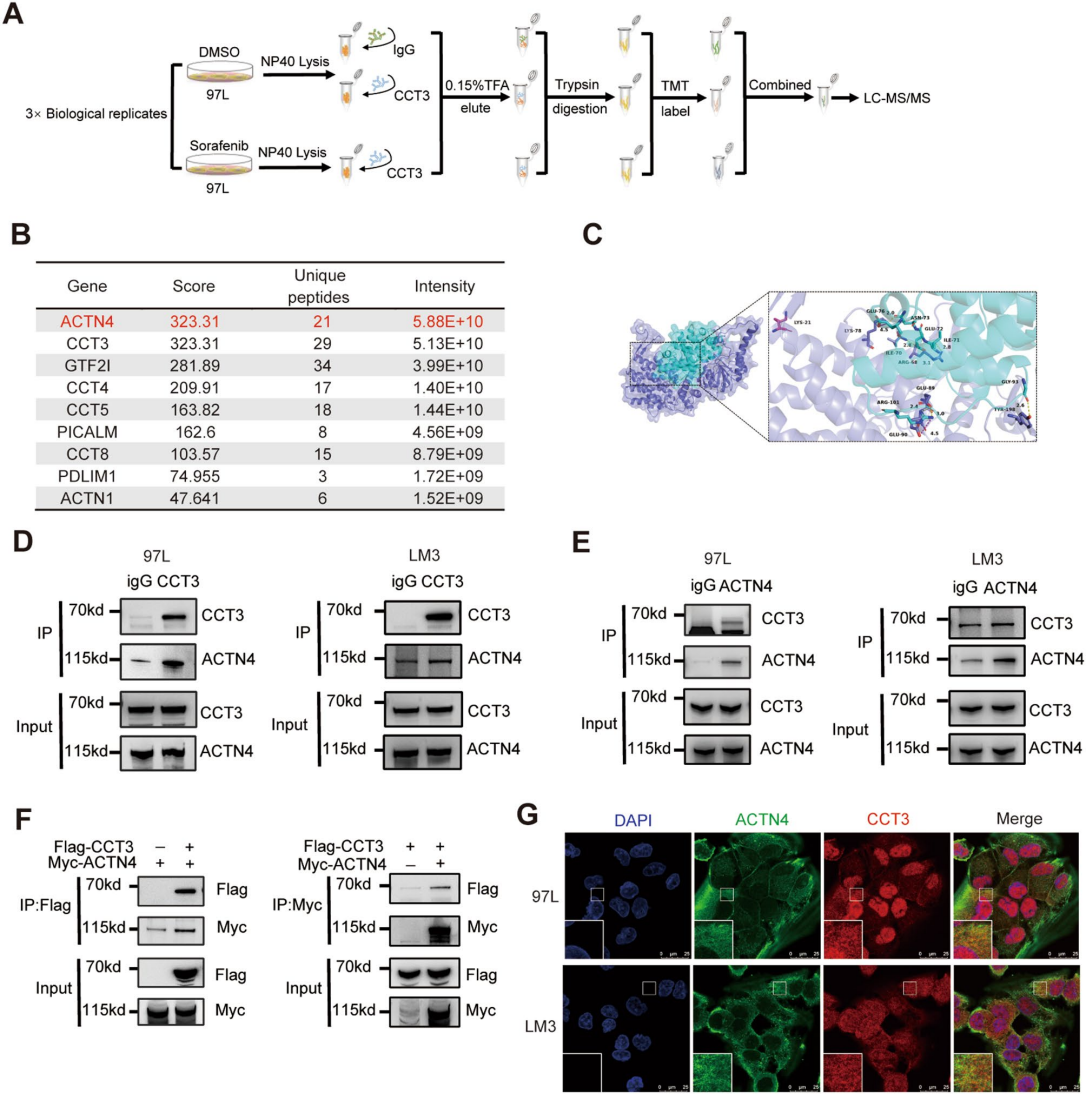
ACTN4 is a interacting protein of CCT3. **A** Flow chart showing the combined use of immunoprecipitation and proteomics to identify the partners of CCT3. **B** List of proteins that may interact with CCT3, sorted by intensity. **C** Molecular docking of the CCT3 (slate cartoon) and ACTN4 (cyan cartoon) interaction complex, with corresponding-colored stick structures representing the binding sites. **D** Analysis of CCT3-binding protein in 97 L and LM3 cells by co-immunoprecipitation. **E** Analysis of ACTN4-binding protein in 97 L and LM3 cells by co-immunoprecipitation. **F** Analysis of CCT3 and ACTN4 binding protein in 293T cells by co-immunoprecipitation. **G** Immunofluorescence staining showing the distribution of CCT3 (red), ACTN4 (green) and DAPI (blue) in indicated cells

To investigate the inhibitory mechanism of ferroptosis by CCT3, we employed co-immunoprecipitation in conjunction with mass spectrometry to identify potential protein interactions with CCT3 (Fig. 4A). The proteins that exhibited a greater pull-down by CCT3 antibodies compared to the IgG isotype control were compiled (Fig. 4B). Among these proteins, ACTN4 displayed the highest protein abundance. Furthermore, the presence of CCT3 and other subunits of the TRiC, namely CCT4, CCT5, and CCT8, were also detected, indicating the successful implementation of our methodology. Notably, we observed a significant enhancement in the binding between CCT3 and ACTN4 upon Sorafenib treatment (Figure. S4A). Using Pymol, the identification and classification of all functional residues were conducted based on their interactions with other proteins. Specifically, the residues LYS78 of CCT3 and ASN73 of ACTN4 were found to form hydrogen bonds, indicating a strong interaction. The CCT3-ACTN4 interaction score was determined to be -557, which is considered favorable (Fig. 4C). To further validate the interaction between CCT3 and ACTN4, endogenous co-immunoprecipitation experiments were performed in 97 L and LM3 cell lines. The results demonstrated that both anti-CCT3 antibodies and anti-ACTN4 antibodies were able to simultaneously pull down CCT3 and ACTN4 (Figs. 4D-E, S4B-C). In addition, exogenous co-immunoprecipitation was performed in 293T cells, revealing that the Flag-CCT3 and Myc-ACTN4 proteins were simultaneously pulled down by the anti-Flag antibody and anti-Myc antibody (Figs. 4F, S4D). Colocalization of CCT3 and ACTN4 was also observed through confocal laser scanning microscopy (Fig. 4G). Consequently, it can be concluded that CCT3 interacts with ACTN4, and the administration of Sorafenib may enhance this interaction.

### The CCT3/ACTN4 axis inhibits ferroptosis by impeding TFRC recycling

**Fig. 5:**
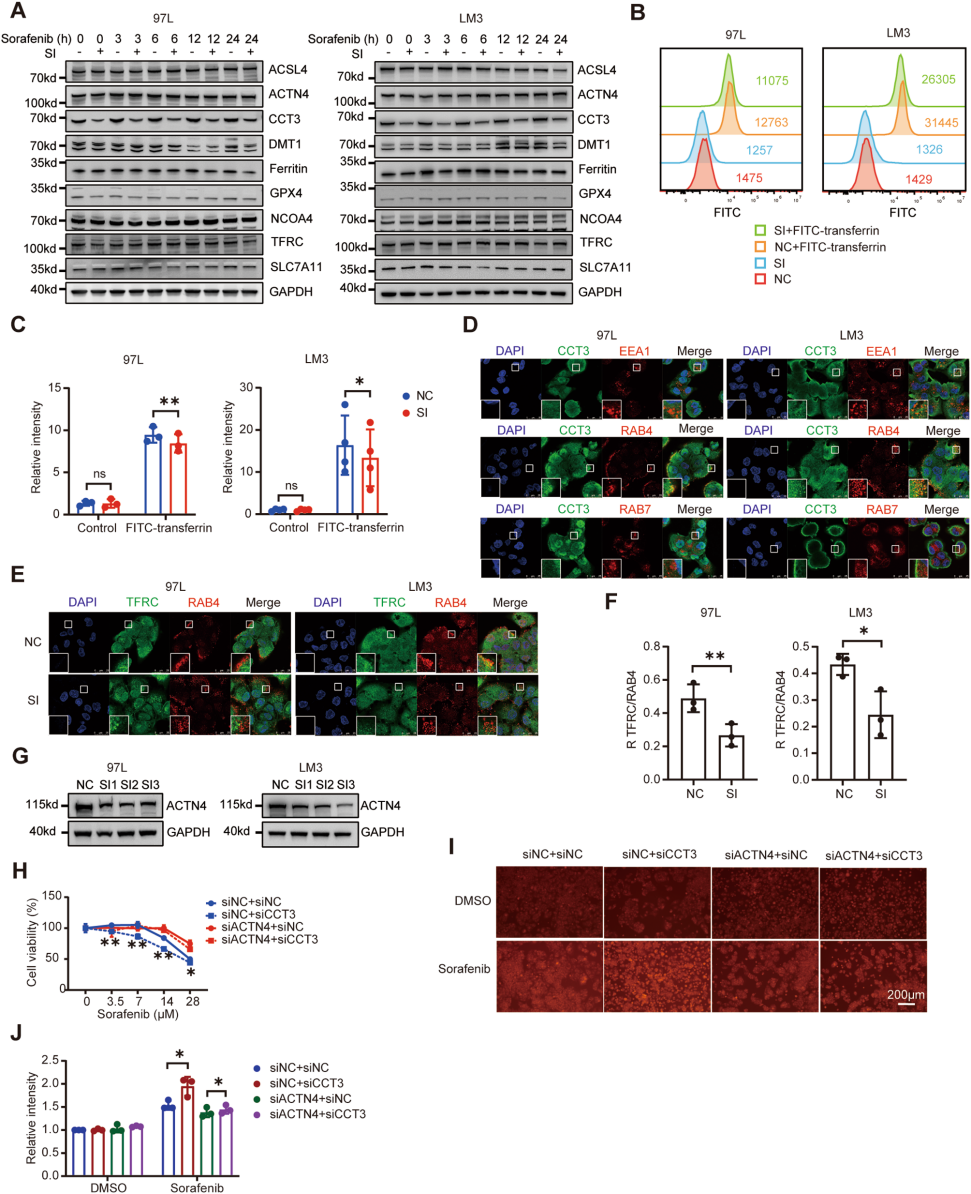
CCT3 inhibits iron endocytosis by impeding TFRC recycling through ACTN4. **A** Western blot analysis of the indicated proteins in control and CCT3-knockdown 97 L and LM3 cells after treated with Sorafenib (7 μM). **B** Indicated cells were incubated in fetal bovine serum free medium for 60 min and then incubated in medium contained 25 μg/ml FITC-transferrin for 15 min. Fluorescence intensity of intracellular FITC was measured by flow cytometry. **C** Histogram showing the statistical results of median FITC fluorescence intensity for indicated groups from three biological replicates. **D** Immunofluorescence staining showing the distribution of CCT3 (green), EEA1 (red), RAB4 (red), RAB7 (red) and DAPI (blue) in indicated cells. **E** Immunofluorescence staining showing the distribution of RAB4 (red), TFRC (green) and DAPI (blue) in control and CCT3-knockdown cells. **F** Pearson’s R correlation value between RAB4 and TFRC in indicated cells. **G** Western blot analysis of the knockdown efficiency of ACTN4 in 97 L and LM3 cells. **H** Cell viability analysis of control (siNC), CCT3-knockdown (siCCT3), and ACTN4-knockdown (siACTN4) 97 L cells following treatment with Sorafenib for 24 h. **I-J** Indicated 97 L cells were treated with Sorafenib (14 μM) for 12 h, and then iron accumulation were measured. Histogram showing the statistical results from three biological replicates

ACTN4 has been previously documented as essential for the efficient recycling of TFRC, which is responsible for maintaining sufficient TFRC levels on the cell membrane to support iron endocytosis [26]. Consequently, we postulated that CCT3 may impede Sorafenib-induced ferroptosis by obstructing iron uptake mediated by ACTN4. To substantiate this conjecture, we initially assessed the impact of Sorafenib and CCT3 knockdown on the expression of proteins associated with ferroptosis. Western blot analysis revealed a reduction in GPX4 levels in 97 L and LM3 cells following Sorafenib treatment, indicating the induction of ferroptosis by Sorafenib. Furthermore, the downregulation of CCT3 did not exhibit any impact on the levels of ACTN4, Ferritin, and TFRC, indicating that CCT3 may potentially interact with ACTN4 to influence its capacity to sustain TFRC recycling, rather than influencing its expression or degradation (Figs. 5A, S4E-F). To validate the influence of CCT3 on iron endocytosis, 97 L and LM3 cells were cultivated in serum-free medium supplemented with FITC-transferrin for a duration of 15 min, followed by removal and fixation at 4% polyformaldehyde, and subsequent analysis of fluorescence intensity using flow cytometry. Unsurprisingly, the fluorescence intensity of FITC in cells with CCT3 knockdown exhibited a significant decrease compared to that in control cells (Fig. 5B-C). The intracellular level of transferrin is known to be influenced by various processes such as endocytosis, recycling, and lysosomal degradation [27, 28]. Consequently, we investigated the specific impact of CCT3 on these processes by utilizing markers for early endosomes (EEA1), recycling endosomes (RAB4), and late endosomes (RAB7) [29–31]. Through immunofluorescent confocal laser microscopy, it was observed that CCT3 co-localized with RAB4, while no co-localization was observed with EEA1 or RAB7 in 97 L and LM3 cells (Fig. 5D). ACTN4 was found to co-locate with EEA1 and RAB4, while TFRC was found to co-locate with EEA1, RAB4, and RAB7, which is consistent with previous research (Fig. S5A-B). To further substantiate the impact of CCT3 on the recycling process of TFRC, we examined the alterations in the co-locational Pearson correlation coefficients between TFRC and endosome markers following CCT3 knockdown. The findings revealed a significant decrease in the co-location between TFRC and RAB4 after CCT3 knockdown, a slight decrease in the co-location between TFRC and EEA1, and no change in the co-location between TFRC and RAB7 (Figs. 5E-F, S5C-F). This finding aligns with previous result indicating that the inhibition of CCT3 leads to an increased rate of TFRC recycling, ultimately resulting in a reduction of TFRC within the recycling endosome. Finally, we conducted a series of representation experiments to demonstrate that ACTN4 and TFRC are indispensable for CCT3 to perform its anti-ferroptosis function. The expression of ACTN4 and TFRC in 97 L and LM3 cells was effectively silenced using siRNA (Fig. 5G, S5G-I). The CCK8 assay revealed that the impact of CCT3 knockdown on Sorafenib sensitivity in HCC cells was no longer observed upon simultaneous knockdown of ACTN4 or TFRC (Figs. 5H, S5J). Furthermore, the observed phenomenon of CCT3 knockdown promoting the accumulation of ferrous ions in Sorafenib-treated cells was not evident after knockdown of ACTN4 or TFRC (Figs. 5I-J, S5K-L). In conclusion, the inhibition of ferroptosis by CCT3 primarily stems from its disruption of ACTN4’s role in TFRC recycling.

### The ferroptosis inhibition function of CCT3 requires deubiquitination at K21

**Fig. 6:**
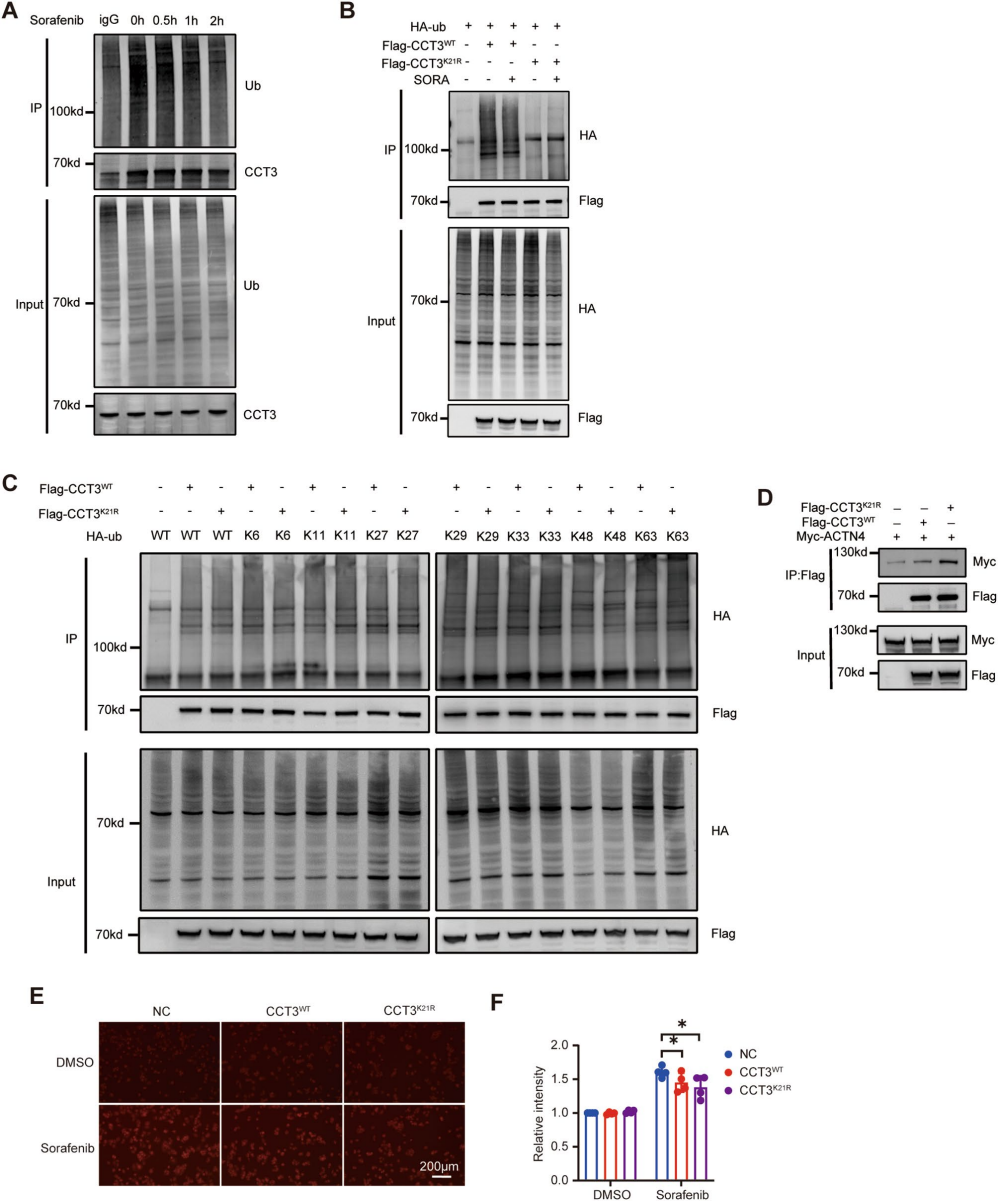
Ubiquitination at K21 was important for CCT3. **A** The 97 L cells were treated with Sorafenib (14 μM) for the indicated times and CCT3 ubiquitination was measured via immunoprecipitation-western blot assay. **B** After overexpression wild-type (CCT3^WT^) or K21R mutant CCT3 (CCT3^K21R^), its ubiquitination was measured in 293T cells treated with DMSO or Sorafenib (14μM) for 30 min. **C** 293T cells were transfected with plasmids expressing indicated wild-type or mutated proteins, CCT3 ubiquitination was measured via immunoprecipitation-western blot assay. K6, K11, K27, K29, K33, K48 and K63 indicates that all lysines except the indicated lysine have been mutated to arginine. **D** Detection of ACTN4 interactions with wild-type or K21R mutant CCT3 in 293T cells. **E-F** 97 L cells overexpressed wild-type or K21R mutant CCT3 were treated with Sorafenib (14 μM) for 12 h, and then iron accumulation were measured. Histogram showing the statistical results from three biological replicates

In previous PTM omics analyses, it was observed that Sorafenib treatment resulted in a decrease in ubiquitination at K21 in CCT3 (Fig. 1D-E). This finding suggests that ubiquitination may play a significant role in CCT3’s ability to inhibit ferroptosis. To further investigate this, a combination of immunoprecipitation and Western blotting analysis was conducted. The results confirmed that Sorafenib treatment reduced the ubiquitination of CCT3 in HCC cells, but did not affect CCT3 at the mRNA or protein levels (Fig. 6A, S6A-B). Additionally, it was observed that Sorafenib did not decrease the ubiquitination of CCT3 when the K21 residue was mutated to arginine (K21R), indicating that Sorafenib primarily affects the ubiquitination of CCT3 at K21 (Figs. 6B, S6C). Using lysine-mutated ubiquitin, our study revealed that only the overexpression of wild type and K6 ubiquitin (where all lysines except lysine 6 are mutated to arginine) resulted in reduced ubiquitination of CCT3^K21R^ compared to CCT3. This suggests that the ubiquitization at K21 of CCT3 is dominated by K6-linked ubiquitination (Figs. 6C, S6D). Based on these experimental findings, we concluded that Sorafenib treatment leads to a decrease in K6-linked ubiquitination at K21 of CCT3. Furthermore, through an exogenous co-immunoprecipitation experiment in 293T cells, we observed that CCT3^K21R^ exhibited stronger binding to ACTN4 compared to CCT3 (Figs. 6D, S6E). The results of molecular docking also showed a strong bond between CCT3^K21R^ and ACTN4 (Figs. S6F). Importantly, the overexpression of both CCT3^K21R^ and CCT3 hindered the accumulation of ferrous ions in Sorafenib-treated 97 L and LM3 cells (Fig. 6E-F, S6G-H). Collectively, these results provide evidence that Sorafenib treatment leads to a reduction in K6-linked ubiquitination in K21 of CCT3, consequently facilitating its association with ACTN4 and its role in suppressing ferroptosis.

### Inhibition of CCT3 enhanced sorafenib’s anti-cancer effect in vivo

**Fig. 7:**
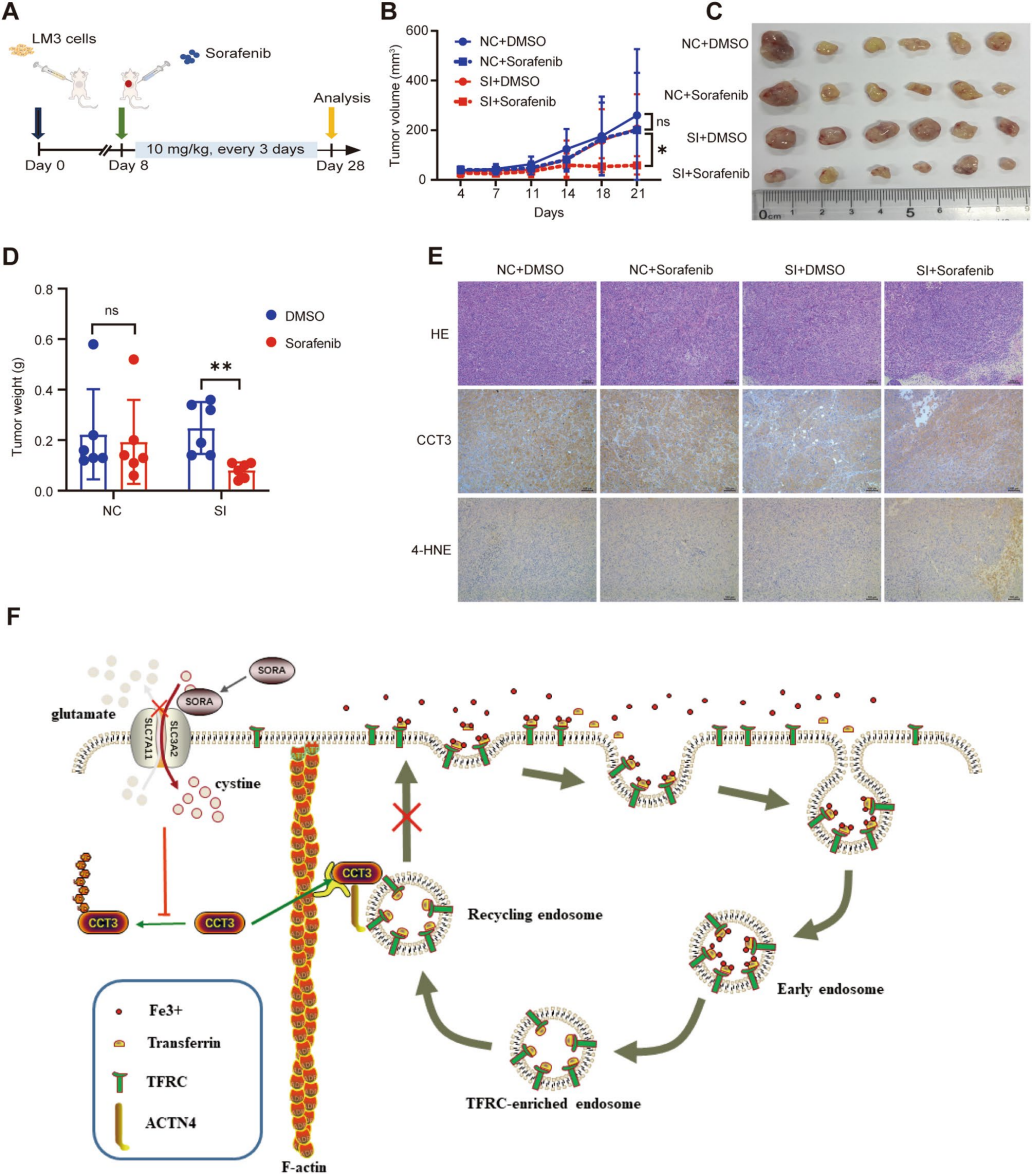
Reducing CCT3 expression enhanced the anti-tumor ability of Sofafenib in vivo. **A** The flow chart points out the main experimental procedures of subcutaneous tumor formation in nude mice. **B** Tumor growth curves, 6 nude mice per group. **C** Isolated subcutaneous tumors from each treatment group are shown. **D** The bar graph shows the weight of the tumor. **E** An immunohistochemistry study of LM3-derived xenograft tumors stained with CCT3 and 4-HNE. **F** A model illustrating the function of CCT3 in inhibiting ferroptosis

We conducted additional research to determine the impact of CCT3 on the effectiveness of sorafenib in an in vivo setting. Nude mice were injected with control and CCT3 knockdown LM3 cells subcutaneously, followed by intraperitoneal injections of either DMSO or sorafenib after one week. The subcutaneous tumors were then collected after three weeks (Fig. 7A). In accordance with our in vitro findings, sustained depletion of CCT3 in conjunction with sorafenib led to a marked decrease in tumor volume and mass (Fig. 7B-D). Surprisingly, the control group exhibited only a modest and statistically nonsignificant decline in tumor size and weight following sorafenib treatment, potentially attributable to the low dosage and infrequent administration of sorafenib in our experimental design. Furthermore, the intensity of 4-HNE in tumor specimens from the CCT3 knockdown cohort surpassed that of the control group post-sorafenib therapy, suggesting heightened oxidative stress within the tumor (Fig. 7E). Based on these results, sorafenib had an enhanced ferroptosis-inducing and tumor suppressive effect when CCT3 was knocked down in vivo.

## Discussion

In 2013, Cong L et al. made the significant discovery that CRISPR arrays possess the capability to encode multiple guide sequences, thereby facilitating the simultaneous editing of multiple sites [32]. As a result, the technique of CRISPR/Cas9 knockout library screening has undergone continuous enhancement and application in the identification of genes associated with the decline in cellular population fitness, encompassing diminished viability, heightened drug susceptibility, and decreased proliferation. Notably, the CRISPR/Cas9 knockout library screening method exhibits the capacity to manipulate nearly the entirety of the genome, encompassing non-coding elements. However, the limitations associated with off-target effects, as well as the presence of heterogeneous and heterozygote knockouts, undermine the dependability of its findings [33]. PTM omics, on the other hand, presents a potent methodology for comprehensively characterizing PTMs at a system-wide level and has found extensive application in the identification of potential drug targets and the elucidation of drug mechanisms [34]. Nevertheless, it is important to note that while PTM omics can identify proteins implicated in drug action, it does not ascertain whether these proteins exert a positive or negative influence on the efficacy of the drug. Therefore, the integration of CRISPR/Cas9 knockout library screening and PTM omics serves to address the limitations of both techniques. Specifically, this study represents an instance of combining CRISPR/Cas9 knockout library screening with PTM omics, leading to the identification of CCT3 as a factor that positively influences HCC resistance to Sorafenib. Furthermore, it was observed that the ubiquitination of CCT3 on K21 decreased following Sorafenib administration.

Our in vitro and in vivo experiments elucidate the underlying mechanism through which CCT3 contributes to Sorafenib resistance in HCC (Fig. 7F). Specifically, Sorafenib treatment diminishes the K6-linked ubiquitination of CCT3 on K21, thereby enhancing the interaction between CCT3 and ACTN4. This interaction hampers the functionality of ACTN4 in facilitating the recycling of TFRC, impeding the endocytotic TFRC from returning to the cell membrane. Consequently, this disruption leads to a reduction in the influx of iron ions into the cell. It can be deduced that CCT3 exerts a negative regulatory influence on intracellular iron ion levels, ultimately inhibiting Sorafenib-induced ferroptosis.

CCT3 typically assumes a barrel-like conformation alongside the other seven members of the CCT family, wherein two rings of subunits enclose a central cavity [35]. This multi-subunit oligomer plays a crucial role in the folding of cytoskeletal proteins and the maintenance of cellular proteostasis [36]. Additionally, CCT3 can also function independently by interacting with YAP and TFCP2 in liver cancer, thereby preventing the PCBP2-induced ubiquitination of these proteins [37]. In the present study, we observed that CCT3 has the ability to bind to ACTN4 and impede its functionality. However, due to the consistent expression of subunits within the CCT complex, altering the expression of one subunit will inevitably impact the expression of other subunits [38, 39]. Consequently, it is challenging to ascertain definitively whether the observed function is solely attributed to CCT3 or the entire CCT complex. Thus, additional investigations are warranted to address this issue. Presently, there is a dearth of literature pertaining to the PTM of CCT3; however, other subunits within the CCT complex, such as CCT2, have been characterized [40]. Our study elucidated that the ubiquitination of CCT3 at K21 resulted in a modification of its function and provided the initial identification of the specific ubiquitin chain type at this site. Additionally, we observed that Sorafenib treatment attenuated this ubiquitination modification, although the precise underlying mechanism necessitates further investigation. Previous reports have highlighted the impact of CCT3 on the prognosis of HCC, primarily focusing on its role in promoting proliferation and inhibiting apoptosis. In contrast, our study innovatively demonstrated that CCT3 can confer resistance to Sorafenib, the frontline therapeutic agent for HCC.

Ferroptosis encompasses multiple metabolic pathways, namely iron metabolism, GSH metabolism, and lipid metabolism. Iron metabolism, in particular, encompasses the processes of iron absorption, storage, and utilization. This aspect of iron metabolism is not only implicated in ferroptosis but also plays a crucial role in normal physiological functions such as heme synthesis and DNA replication [41]. Cellular iron absorption occurs through two mechanisms: mediated by TFRC, which transports iron bound to transferrin, or facilitated by solute carrier family 39 member 14 (SLC39A14), which transports iron that is not bound to transferrin [42]. The observation of TFRC overexpression in certain tumor cells and the inhibitory effect of TFRC deletion on erastin-induced ferroptosis have been documented [43, 44]. Previous studies have primarily focused on the regulation of ferroptosis by TFRC through its differential expression, yet the present study found no significant alteration in TFRC expression following Sorafenib treatment [45]. Considering the involvement of TFRC-mediated iron uptake via endosomal pathways, it was hypothesized that Sorafenib and CCT3 might impact this process [46]. Moreover, previous research has indicated that ACTN4, the specific protein targeted by CCT3 as identified in this investigation, plays a role in the process of endocytosis [47–49]. Consequently, we proceeded to investigate the impact of CCT3 on the endocytosis of TFRC by conducting a series of experiments involving mechanisms and representations. Our findings unequivocally demonstrate that CCT3 hinders the recycling of endosomes containing TFRC.

In conclusion, our study employed PTM omics and CRISPR/Cas9 knockout library screening to systematically identify CCT3 as a significant gene associated with Sorafenib resistance. Notably, our findings demonstrate that CCT3 hinders TFRC recycling through its interaction with ACTN4, and this regulatory mechanism is governed by K6-linked ubiquitination on the K21 sites. This study holds theoretical significance by uncovering novel functions of chaperones and a previously unknown negative feedback mechanism of ferroptosis. Furthermore, this study holds significant clinical implications in the realm of guiding therapy treatments for HCC and addressing the issue of Sorafenib resistance. One potential avenue to overcome this resistance involves the development of drugs that can effectively inhibit the interaction between CCT3 and ACTN4. Previous investigations have demonstrated that ferroptosis can hinder tumor immunity, resulting in paradoxical effects [50]. Consequently, it is imperative to conduct further research to investigate the impact of CCT3 on the tumor immune microenvironment.

### Methods

#### Reagents

Anti-CCT3 antibody (proteintech, 10571-1-AP), anti-TFRC antibody (proteintech, 10084-2-AP), anti-ubiquitin antibody (Cell Signaling Technology, 3936), anti-GPX4 antibody (proteintech, 14432-1-AP), anti-NCOA4 antibody (abcam, ab86707), anti-Myc antibody (proteintech, 16286-1-AP), anti-ACTN4 antibody (proteintech, 19096-1-AP), anti-ACSL4 antibody (Santa Cruz Biotechnology, sc-271800), anti-Ferritin antibody (Santa Cruz Biotechnology, sc-376594), anti-SLC7A11 antibody (Cell Signaling Technology, 12691 S), anti-Flag antibody (Bioss, bsm-33346 M), anti-GAPDH antibody (Fdbio, FD0063), anti-DMT1 antibody (proteintech, 20507-1-AP), anti-HA antibody (Santa Cruz Biotechnology, sc-7392), protein A/G (Santa Cruz Biotechnology, sc-2003), FITC-transferrin (Rockland Immunochemicals, 009-0234), BODIPY 581/591 C11 (ThermoFisher, D3861), FerroOrange (Dojindo, F374), Sorafenib (CSNpharm, CSN10381), Z-VAD-FMK (Selleck, S7023), Ferrostatin-1 (Selleck, S7243), Necrostatin-1 (Selleck, S8037), PTMScan Phospho-Tyrosine Rabbit mAb (P-Tyr-1000) Kit (Cell Signaling Technology, 8803), PTMScan Ubiquitin Remnant Motif (K-ε-GG) Kit (Cell Signaling Technology, 5562), PTMScan Acetyl-Lysine Motif [Ac-K] Kit (Cell Signaling Technology, 13416), GSH/GSSG detection assay (Beyotime, S0053), lipofectamine (ThermoFisher, 56532), Neofect (NEOFECT, TF20121201).

#### Cell culture

The non-SORA resistant 97 L and LM3 cell lines were procured from the Meisen Chinese Tissue Culture Collections (MeisenCTCC). These cell lines were cultivated in Dulbecco’s modified Eagle’s medium (DMEM), supplemented with 10% fetal bovine serum (FBS) and 1% penicillin/streptomycin, at a temperature of 37 °C. To ensure their authenticity, we conducted short tandem repeat profiling on all cell lines and also tested them for mycoplasma contamination.

#### Western blot

The cells were lysed using RIPA lysis buffer supplemented with a protease inhibitor cocktail and PR-619 on ice for a duration of 20 min. After centrifugation at 15,000 g for 15 min at 4 °C, the resulting supernatants were collected and individually mixed with 5 × sodium dodecyl sulfate (SDS) loading buffer. Subsequently, a 4–10 μL sample of protein was loaded onto 4–12% gradient Bis-Tris gels and transferred onto polyvinylidene difluoride membranes for blotting. After being blocked with a 1 × blocking solution at room temperature for one hour, the membranes were subjected to overnight incubation at 4 °C with various primary antibodies (diluted at 1:500-1:1000). Subsequently, the membranes were washed three times with 1 × tris buffered saline tween (TBST) and then incubated with HRP-conjugated secondary antibodies for two hours at room temperature. Following another three washes with 1 × TBST, the protein lanes were visualized using enhanced chemiluminescence and analyzed utilizing the Chemiluminescent Imaging System.

#### Cell viability assay

Cell viability was assessed by employing the Cell Counting Kit-8 (CCK8) in accordance with the guidelines provided by the manufacturer. The cells were seeded into 96-well plates at a density ranging from 5 to 10 × 10^3^ cells per well and subsequently subjected to Sorafenib treatment. Following a duration of 24–48 h, the cell culture medium was substituted with fresh DMEM supplemented with 10% CCK8 solutions, and the plates were reintroduced into a 37 °C incubator for a period of 2 h. The absorbance at 450 nm was then quantified using a spectrophotometer.

#### Cell apoptosis assay

Cell apoptosis was assessed utilizing the Annexin V/propidium iodide (PI) apoptosis detection kit in accordance with the manufacturer’s guidelines. Both adherent and nonadherent cells in the supernatant were harvested and subjected to two washes with phosphate-buffered saline (PBS). Subsequently, a 100 μL cell suspension, resuspended at a density of 1 × 10^6^ cells/ml, was treated with 5 μL of Annexin V and PI staining solution. The resulting FITC-conjugated Annexin V and DNA-bound PI were then analyzed using flow cytometry.

#### RNA interference and plasmid transfection

The transfection of siRNA and plasmid was conducted using lipofectamine 3000 or Neofect in accordance with the manufacturer’s protocols. Cells were passaged at the appropriate density 12 h prior to transfection, and transfection reagents, along with siRNA or plasmid, were separately mixed with opti-MEM and allowed to incubate for 5 min. The two mixtures were then combined and left to incubate for an additional 15 min before being added to the medium. The efficacy of knockdown or overexpression was assessed 48 h post-transfection through Western blot or qPCR analysis. Supplementary Table 3 contains a compilation of siRNA sequences.

#### Immunoprecipitation analysis

The 97 L, LM3, and 293FT cell lines were harvested and lysed at 4 °C using 0.5% NP40 for CO-immunoprecipitation or RIPA lysis buffer for immunoprecipitation. Following centrifugation at 15,000 g for 20 min at 4 °C, a supernatant was obtained and incubated overnight at 4 °C with gentle shaking, using either a specific antibody or an isotype control IgG. After the addition of protein A/G agarose beads, incubation was continued for an additional 2 h. The beads were then washed five times with PBS before being either boiled in SDS loading buffer or eluted using 0.15% trifluoroacetic acid (TFA).

#### Immunofluorescence analysis

In this study, 97 L and LM3 cells were seeded and treated in 24-well plates with cell climbing slices. The cells in the cell climbing slices were fixed using a 4% polyformaldehyde solution, and a membrane-breaking solution was employed to disrupt the cell membrane. Subsequently, the cells were blocked for 15 min using a blocking solution and then incubated overnight at 4℃ with primary antibodies. After washing the cells three times with a wash buffer, they were incubated with Alexa Fluor 555 and Alexa Fluor 488 conjugated secondary antibodies for 1 h at room temperature. Finally, DAPI anti-quenching sealant was applied to the slides, and confocal laser microscope imaging was used to capture the images.

#### Transferrin endocytosis assay

The cells in a 12-well plate were subjected to two washes with serum-free DMEM and subsequently incubated with the same medium at a temperature of 37 °C for a duration of 1 h. Following this, a serum-free DMEM medium containing 25 μg/ml of FITC-transferrin was introduced, and the cells were further incubated at 37 °C for a period of 15 min. The adherent cells were dissociated using 0.25% trypsin-EDTA, fixed with 4% paraformaldehyde at room temperature for 20 min, and subjected to two washes with PBS. The intracellular FITC-transferrin signals were then quantified using a flow cytometer equipped with FITC detection settings.

#### GSH/GSSG detection assay

The intracellular levels of glutathione (GSH) and oxidized glutathione (GSSG) were assessed using GSH/GSSG assay kits following the manufacturer’s instructions. In summary, cells in a 24-well plate were harvested and homogenized with protein removal agent M solution. Subsequently, the samples underwent two cycles of freezing and thawing using liquid nitrogen and 37 °C water baths. After a 5-minute incubation on ice, the supernatant was obtained by centrifugation at 10,000 g for 10 min for the quantification of glutathione and oxidized glutathione.

#### Transmission electron microscopy

The cells were subjected to fixation for a duration of 24 h using a 2.5% glutaraldehyde solution. Following three washes with PBS, the cells were exposed to a 1% osmic acid solution for 1 h, followed by treatment with a 2% uranium acetate solution for 30 min. Subsequently, the sample underwent dehydration and embedding processes, enabling the creation of ultrathin sections. Digital images were obtained using transmission electron microscopy at magnifications of 2000× and 8500×.

#### Colony formation assay

The LM3 and 97 L cells were suspended in a solution of 10% FBS and DMEM. A seeding density of 1000 cells per well was utilized in six-well plates, followed by treatment with Sorafenib at the specified concentrations. The cells were maintained in a humidified incubator at a temperature of 37℃, and the medium was replaced every 2 to 3 days. After a duration of two weeks, the cells were fixed using a 4% solution of paraformaldehyde for a period of 20 min. Subsequently, the colonies were stained with a 0.1% solution of crystal violet for 20 min and washed with PBS three times. The colonies were then photographed and quantified using ImageJ software.

### Iron staining

Intracellular labile iron was assessed utilizing the metal sensor FerroOrange. In brief, a total of approximately 5 × 10^4^ cells were seeded per well in a 24-well plate, and the designated treatments were administered to the cells on the subsequent day. Following a 12-hour incubation period, FerroOrange was introduced at a dilution of 2500 times and incubated at a temperature of 37 °C for a duration of 10 min, after which it was captured via fluorescence microscopy. The estimation of intracellular labile iron was determined based on the intensity of yellow fluorescence and quantified using imageJ software.

#### Lipid ROS assay

Intracellular non-peroxidized lipids were assessed by employing the BODIPY 581/591 C11 dye. To initiate the experiment, approximately 1 × 10^5^ cells were seeded per well in a 12-well plate, and the designated treatments were administered to the cells on the subsequent day. Following a 24-hour incubation period, BODIPY 581/591 C11 was introduced and allowed to incubate at a temperature of 37 °C for a duration of 15 min. Subsequently, the cells were fixed with a 4% paraformaldehyde solution, and the non-peroxidized lipids were evaluated using flow cytometry.

#### Molecular docking

The crystal structures of TCPG (7LUM) and ACTN4 (6OA6) were obtained from the Protein Data Bank [51, 52]. The protein was prepared using AutoDockTools-1.5.7, with manual removal of water molecules and addition of polar hydrogen [53]. Protein-protein docking was conducted using the Docking Web Server (GRAMM) [54, 55]. The resulting protein-protein complex underwent manual optimization by AutoDockTools-1.5.7 to eliminate water and add polar hydrogen. Finally, PyMOL was utilized to predict the protein-protein interactions and generate the corresponding figure.

#### Protein extraction and digestion

The 97 L and LM3 cells were harvested and lysed in freshly prepared lysis buffers containing 8 M urea, 150 mM NaCl, 50 mM Tris-HCl pH 8.0, 1 × phosphorylase inhibitor, 1 × deacetylase inhibitor, 50 mM (2,6-diamino-5-thiocyanatopyridin-3-yl) thiocyanate (PR- 619), and 1× protease inhibitor cocktail at a temperature of 4 °C for a duration of 20 min. The protein concentration of the supernatant was estimated using the bicinchoninic acid protein assay (BCA). Protein reduction was achieved by treating with dithiothreitol for 45 min at a temperature of 30 °C, followed by carbamidomethylation using Iodoacetamide for 30 min at room temperature. Subsequently, the solvent was substituted with 50 mmol/L ammonium bicarbonate using a Zeba Spin Desalting Column or Ultrafiltration centrifugal tube. The samples were subjected to overnight digestion at 37 °C, employing a trypsin-to-substrate ratio of 1:40 (wt/wt) and agitation at 150 rpm. The peptides were acidified with trifluoroacetic acid (TFA), desalted through C18 Sep-Pak SPE cartridges, and subsequently subjected to vacuum drying.

#### Enrichment of PTM peptides

Proteomics analyses were conducted to investigate acetylation, phosphorylation, and ubiquitination. Digestive peptides were resuspended in ice-cold 1 × IAP buffer and incubated with cross-linked antibody beads for a duration of two hours at 4 °C. The elution of peptides was achieved using 0.15% TFA after three rounds of soft-washing with ice-cold 1 × IAP buffer and two rounds with ice-cold ultrapure water. The eluate was completely dried through vacuum centrifugation and subsequently utilized for subsequent analysis.

#### Tandem mass tag (TMT) isobaric labeling

The TMT Label Reagents were equilibrated at room temperature prior to use and subsequently dissolved in anhydrous acetonitrile for a duration of 5 min, with intermittent vortexing. A peptide sample was dissolved in a solution containing 100 mmol/L of tetraethylammonium bromide at a pH of 8.0. Following this, the TMT Label Reagent was added to the peptide sample and incubated at room temperature for a period of one hour. To halt the reaction, all samples were treated with 5% hydroxylamine for 15 min and combined into a single tube. The TMT-labeled peptides were then dried using vacuum centrifugation after desalting with a C18 stage tip that was prepared in-house.

#### Strong anion exchange (SAX) fractionation

Empore Anion-SR Extraction Disks were utilized for the purpose of performing SAX chromatography using tip columns. The SAX tip, which was prepared in-house, underwent conditioning and equilibration with 100 μL of acetonitrile (ACN), followed by 100 μL of SAX B, 200 μL of SAX C, and finally 100 μL of SAX A. Mixtures containing PTM peptides labeled with TMT were reconstituted in 100 μL of SAX A and subsequently loaded onto the previously treated SAX tips. Upon loading the SAX tips with 100 μL of SAX buffers 1, 2, 3, 4, and 5, the resulting eluates were designated as SAX fractions 1, 2, 3, 4, and 5, respectively. Supplementary Table 4 provides a comprehensive list of the formulations for the SAX reagents.

#### Liquid chromatography–tandem mass spectrometry (LC-MS/MS) analysis

The peptide samples obtained after SAX fractionation underwent desalting and vacuum evaporation, followed by resuspension in a solution containing 2% ACN and 0.1% formic acid (FA). LC-MS/MS analysis was conducted using a Thermo Fisher Scientific Orbitrap Exploris 480 mass spectrometer coupled to an UltiMate NCS-3500RSC system. Gradient elution was performed at a flow rate of 450 nL/min, with linear gradients lasting for a total of 120 min. Specifically, the solvent composition was as follows: 3-10% solvent B from 1 to 5 min, 10-26% solvent B from 6 to 90 min, 26-38% solvent B from 91 to 110 min, 38-80% solvent B from 111 to 115 min, and 80% solvent B from 116 to 120 min. The MS spectra were obtained with a resolution of 60,000, covering a mass range of 400–1200 m/z, and utilizing a normalized automatic gain control (AGC) target of 300%. For the acquisition of MS2 spectra, a resolution of 15,000 was employed, along with a higher energy collision induced dissociation (HCD) collision energy set at 35%. The isolation window was set at 1.6 m/z, while the dynamic exclusion window lasted for a duration of 30 s.

#### Protein identification and quantification

The identification and quantification of proteins were conducted using MaxQuant (version 1.6.2.10). The MS2-based TMT18plex quantification was employed for the whole cell proteome, while the MS2-based TMT10plex quantification was utilized for the acetylome, phosphoproteome, and ubiquitinome. The human UniProtKB served as the search database, with the automatic reverse database and known contaminants employed as decoys. The carbamidomethyl-cysteine modification was designated as a fixed modification, while the N-terminal acetylation and methionine oxidation modifications were considered as variable modifications. In addition, the variable modifications encompassing the acetylome, phosphoproteome, and ubiquitinome were incorporated. The upper limit for modifications per peptide was established at five. The specific enzyme employed was trypsin, with a tolerance of two missed cleavages per peptide. Any parameters not explicitly mentioned were assigned the default settings of Maxquant.

#### Animal experiment

Four-week-old male nude BALB/c mice that were specific pathogen-free were procured from the Zhejiang Laboratory Animal Center in Hangzhou, Zhejiang, China, and were housed in accordance with the regulations for laboratory animal care established by Zhejiang University in 2009. Nude mice were randomly allocated into four groups, each consisting of six mice. A subcutaneous tumor-bearing model was established by inoculating 5 × 106 LM3 cells in 100 μL matrigel into the subcutaneous tissue of the right hind limbs of the nude mice. The body weight and tumor volume of the mice were monitored every 3–4 days, with tumor volume calculated using the formula 0.5 × length × width2. Following the subcutaneous cell injection for one week, SORA (10 mg/kg, dissolved in 5% DMSO + 45% PEG400 + 50% saline) was administered intraperitoneally every 3 days.

#### Bioinformatics analysis

The functional annotation enrichment analysis for proteins with altered PTM or expression was conducted using the clusterProfiler package in R [56]. The FerrDb database was utilized to obtain the Ferroptosis related pathway in the GSEA analysis [57]. Additionally, the consensus motifs of the PTM were examined by analyzing sequences within − 15 and + 15 amino acids of the PTM sites, and the resulting amino acid sequence diagram was generated using the ggseqlogo package in R.

### Statistical analysis

The statistical and bioinformatics data were predominantly analyzed using the R framework (version 4.3.0) and GraphPad Prism 9. Unless specified otherwise, the experiments were conducted independently in triplicates, and the data is presented as mean ± SD. All statistical significance was determined using a two-tailed, Student’s t-test, unless otherwise stated. A *P*-value of less than 0.05 was considered statistically significant (^ns^*P* ≥ 0.05; ^✱^*P* < 0.05, ^✱✱^*P* < 0.01).

### Electronic supplementary material

Below is the link to the electronic supplementary material.

Supplementary Material 1

Supplementary Material 2

Supplementary Material 3

Supplementary Material 4

Supplementary Material 5

## Data Availability

The mass spectrometry proteomics data deposited to the ProteomeXchange Consortium via the iProX partner repository has been assigned the dataset identifier PXD045576 [58, 59]. Supplementary information containing additional data supporting our findings is also provided.
