# Supplementary material for: CCT3/ACTN4/TFRC axis protects hepatocellular carcinoma cells from ferroptosis by inhibiting iron endocytosis
Source: J Exp Clin Cancer Res. 2024 Aug 29;43:245. doi: 10.1186/s13046-024-03169-7 (PMC11360757; doi:10.1186/s13046-024-03169-7)
Supplement: Supplementary file 5 — Supplementary Material 5 [file 13046_2024_3169_MOESM5_ESM.docx]

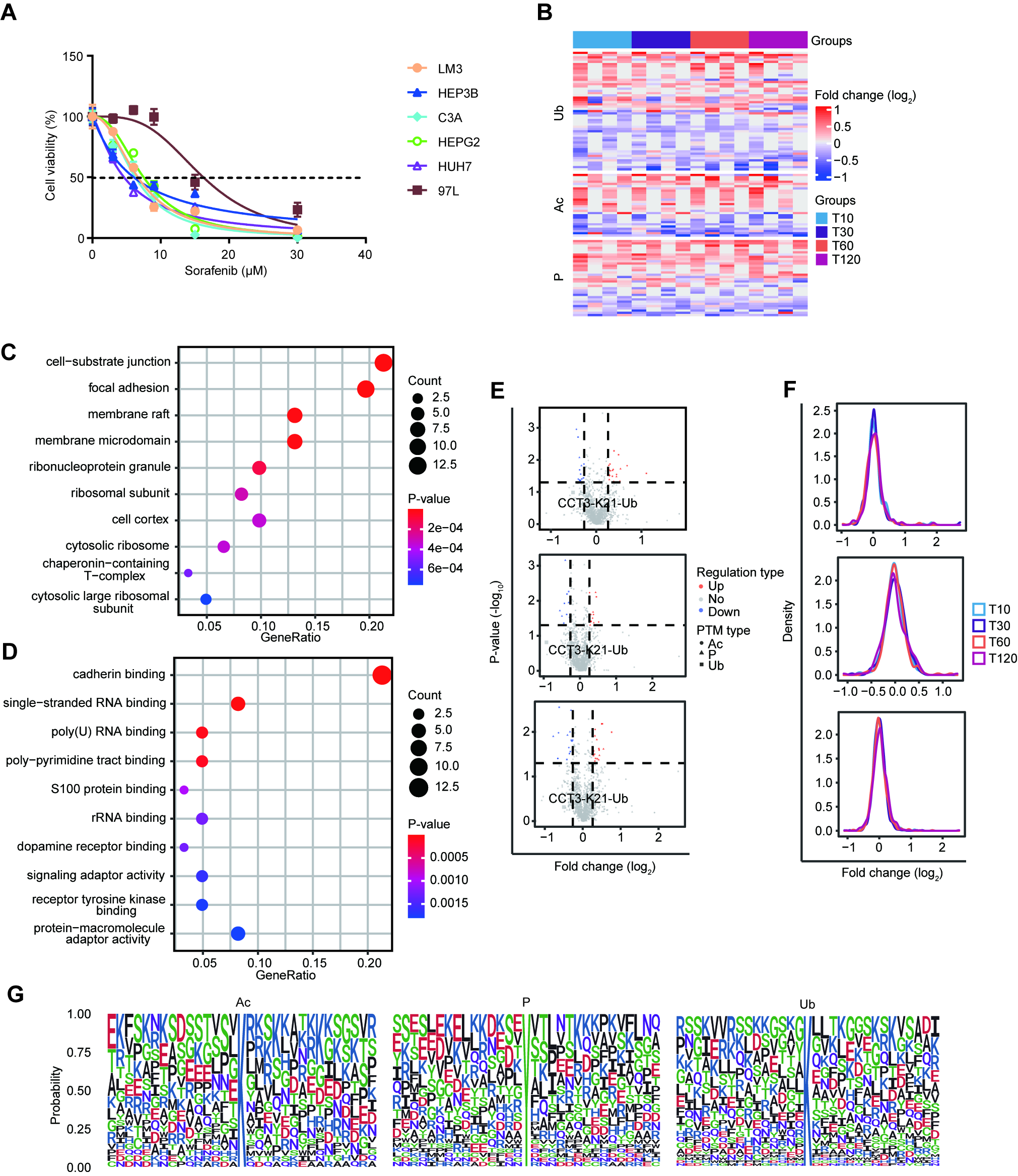


**Fig. S1 PTM omics for 97L cells under Sorafenib treatment. A** Sorafenib drug sensitivity curves in different liver cancer cell lines. **B** Heat maps showed PTM sites with significant regulated in ubiquitination, acetylation and phosphorylation in 97L cells after Sorafenib treatment. **C-D** GO enrichment analysis (cell component and molecular function) of proteins containing regulated PTM site after Sorafenib treatment. **E** Volcano plot showing regulated PTM sites in 97L cells treated with Sorafenib for 10 min, 30 min, and 2 h. **F** Density distribution map of acetylation, phosphorylation and ubiquitination sites after Sorafenib treatment. “T10” means “10 min vs 0 min”. **G** iceLogo plots showing the difference of amino acid frequency at positions flanking the Sorafenib-regulated acetylation, phosphorylation and ubiquitination sites.


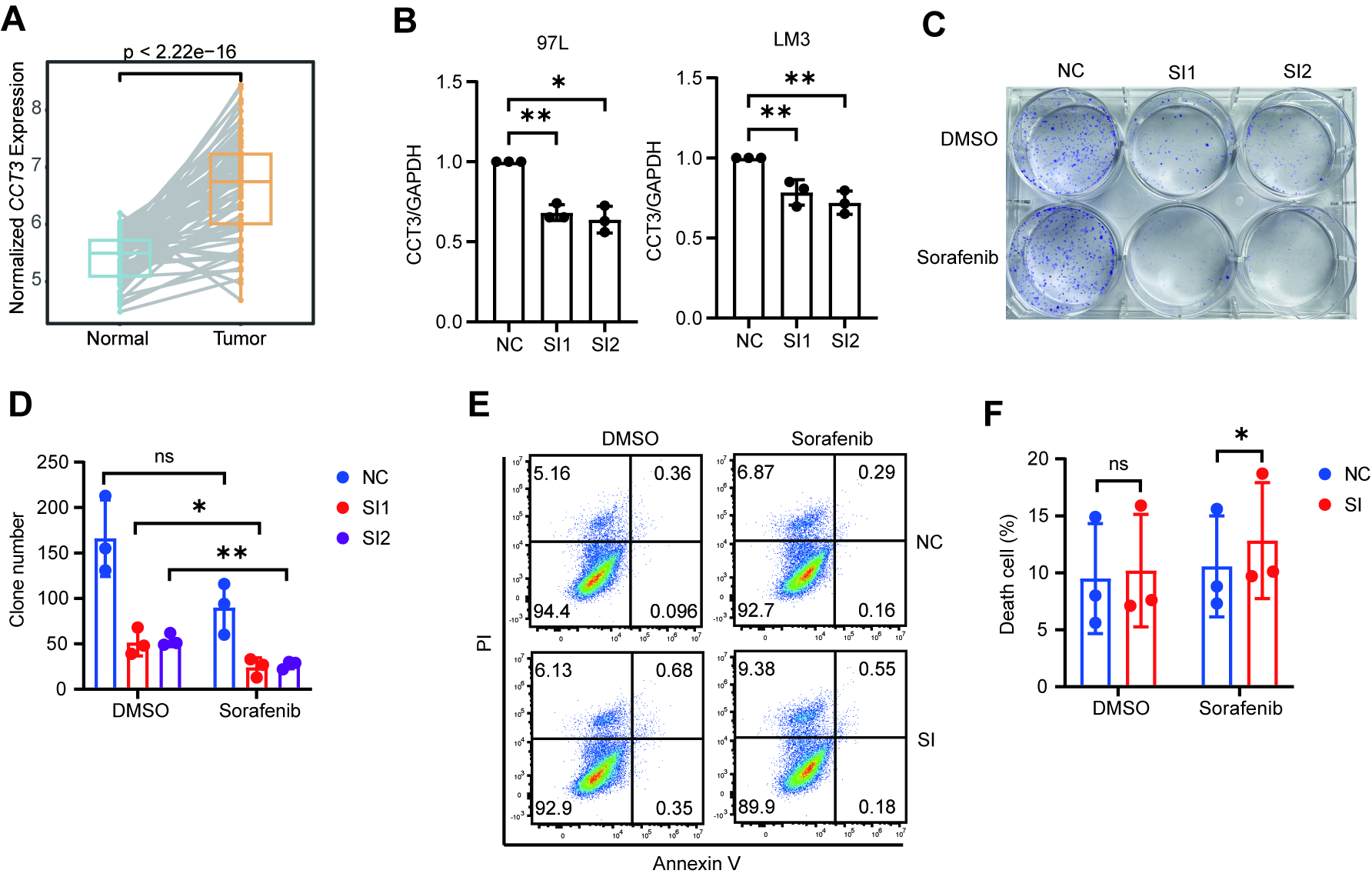
 **Fig. S2 The inhibition of CCT3 sensitized HCC cells to Sorafenib. A** Box plot of CCT3 expression levels in hepatocellular carcinoma tissue and corresponding adjacent non-cancerous tissue. **B** The expression of CCT3 in indicted groups detected by Western blot were quantified. Statistical analysis was performed from three biological replicates. **C-D** Clone formation capacity of control and CCT3-knockdown LM3 cells following treatment with DMSO or 2 µM Sorafenib. Statistical analysis was performed from three biological replicates. **E** Apoptosis of indicted LM3 cells treated with DMSO or 7 µM Sorafenib for 24 h were performed using Annexin V/PI staining. **F** Histogram showing the statistical results of apoptosis from three biological replicates.


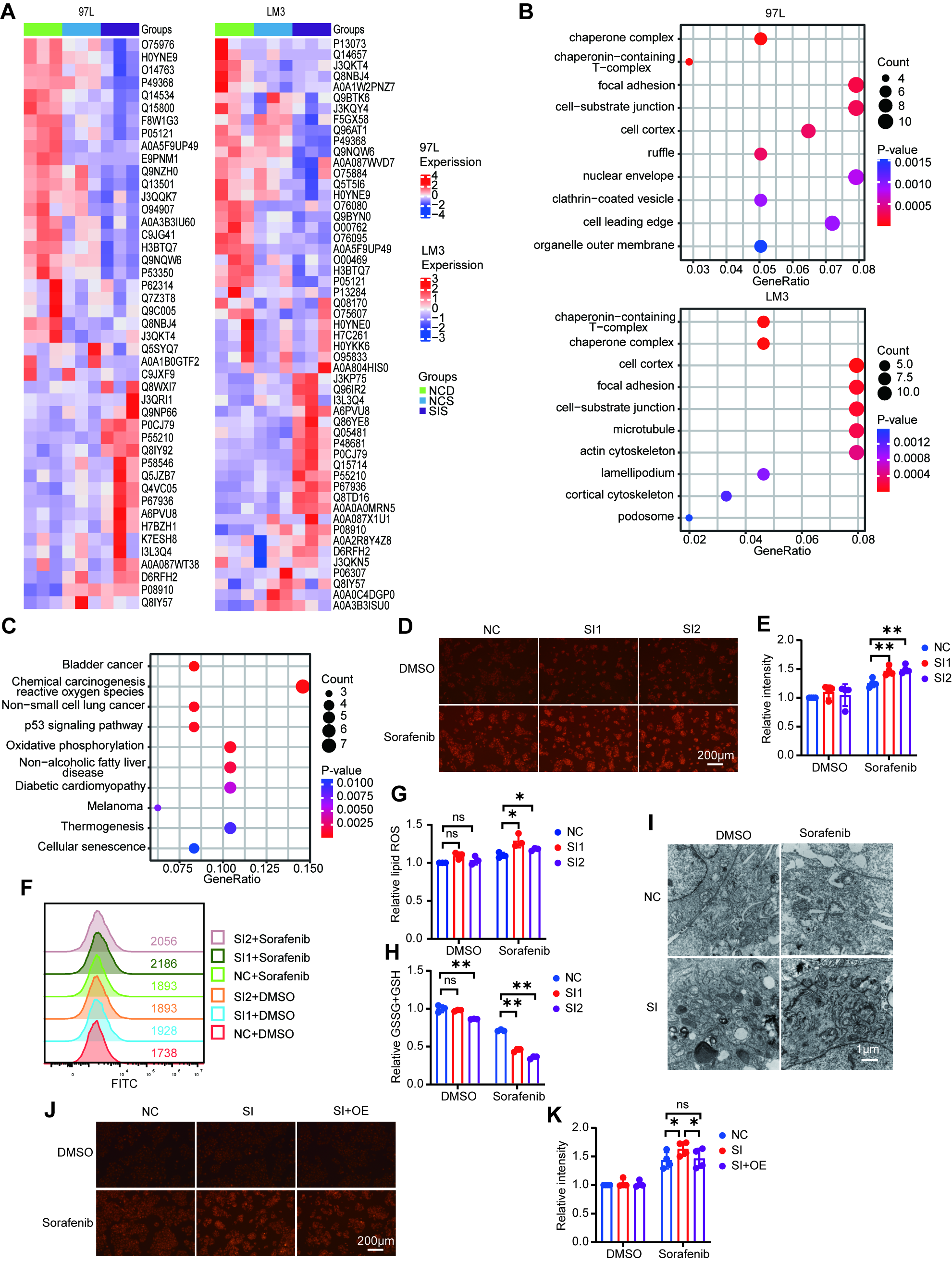
 **Fig. S3 The inhibition of CCT3 promotes ferroptosis in HCC cells treated with Sorafenib. A** Heat map showing the differential gene expression between different groups (NCD: control + DMSO, NCS: control + Sorafenib, SIS: CCT3 knockdown + Sorafenib) in indicated cells. **B** GO enrichment analysis (cell component) of differentially expressed protein between control and CCT3-knockdown cells. **C** KEGG pathway enrichment analysis of differentially expressed protein between DMSO and Sorafenib treatment LM3 cells. **D-E** The accumulation of iron in control and CCT3-knockdown LM3 cells were detected using FerroOrange after treated with DMSO or Sorafenib (14 µM) for 12 h. Statistical results of iron accumulation were from four biological replicates. **F-G** LM3 cells were treated with DMSO or Sorafenib (14 µM) for 24 h, and then lipid hydroperoxides were measured. Statistics for the median Fluorescence intensity of oxidized BODIPY dyes were carried out on three biological replicates. **H** LM3 cells were treated with Sorafenib at 14 µM for 12 h, and the intracellular glutathione (GSH) level were assayed. **I** Transmission electron microscopy was used to examine the morphology of indicated LM3 cells after 24 hours of treatment with Sorafenib (14 µM). **J-K** Indicated LM3 cells were treated with Sorafenib (14 µM) for 12 h, and then iron accumulation were measured and statistical analysis.


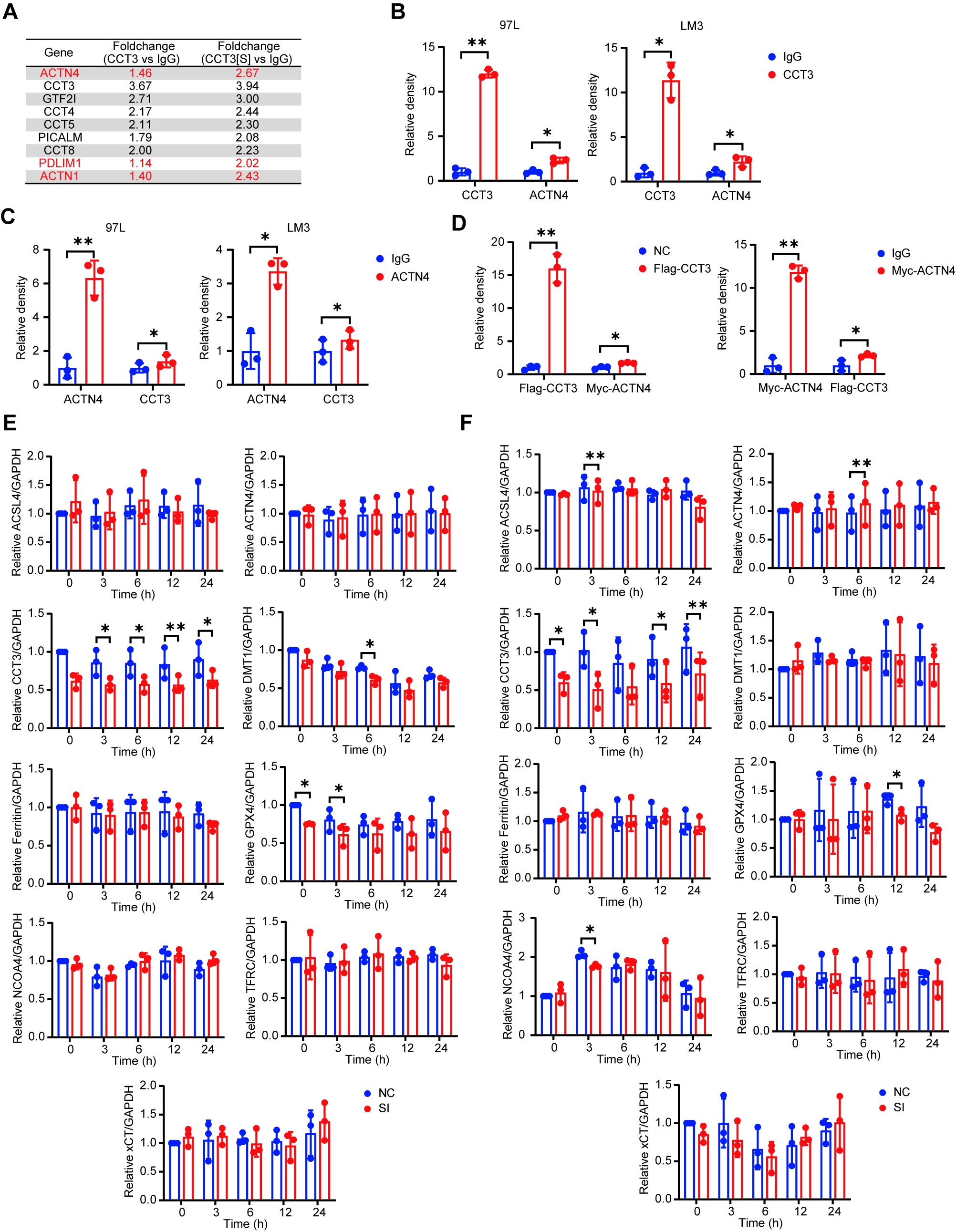
 **Fig. S4 An interacting protein of CCT3 is ACTN4. A** List of proteins with significant changes between indicated groups. **B** Quantitative and statistical analysis of western blot result of CCT3-binding protein in 97L and LM3 cells. **C** Quantitative and statistical analysis of Western blot result of ACTN4-binding protein in 97L and LM3 cells. **D** Quantitative and statistical analysis of western blot result of CCT3-binding and ACTN4-binding protein in 293T cells. **E-F** The expression of CCT3, ACTN4 and protein associated with ferroptosis after Sorafenib treatment in 97L and LM3 cells were quantified and analyzed. All statistical analysis was performed from three biological replicates.


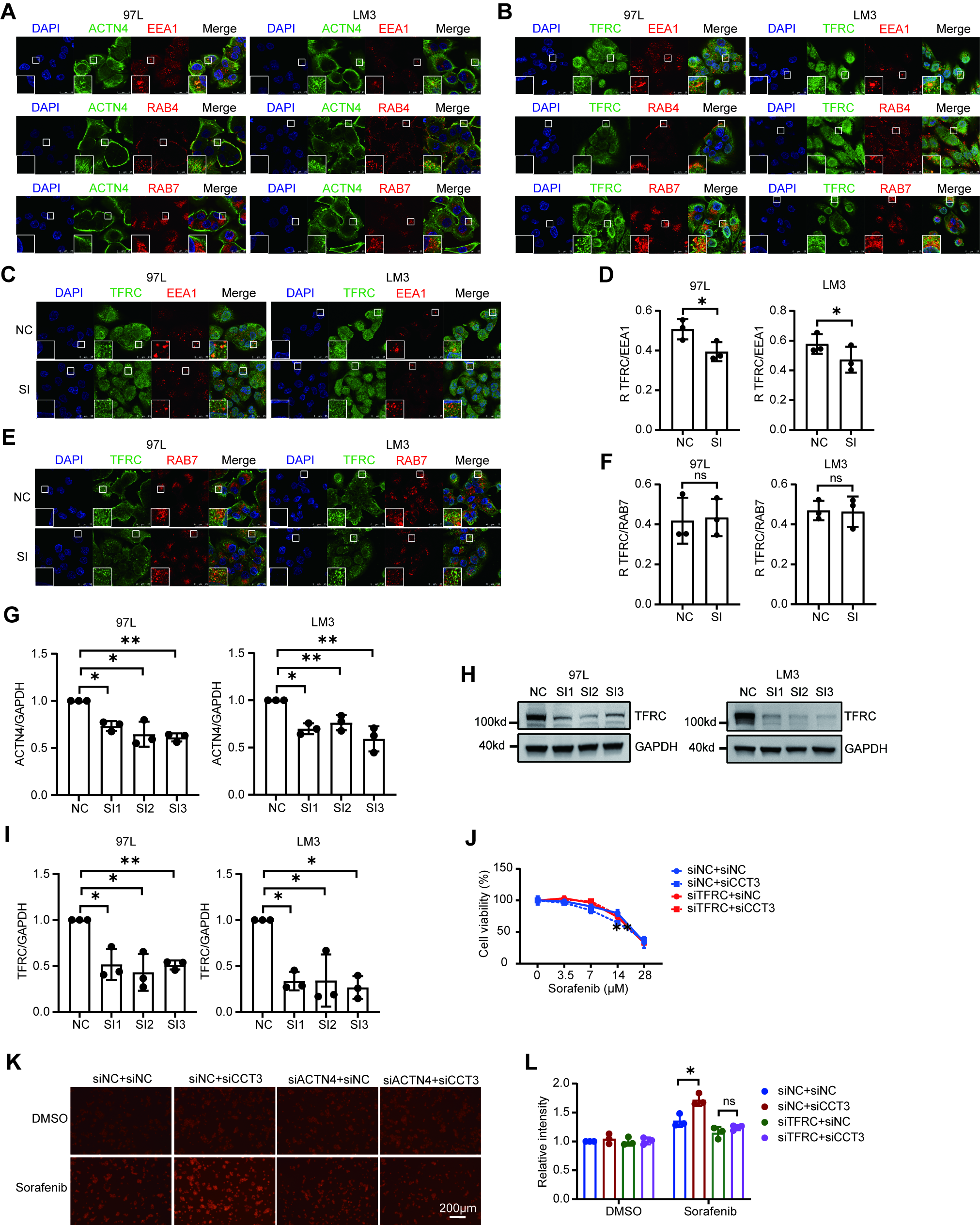
 **Fig. S5 Iron endocytosis is inhibited by CCT3 by impairing TFRC recycling through ACTN4. A** Immunofluorescence staining showing the distribution of ACTN4 (green), EEA1 (red), RAB4 (red), RAB7 (red) and DAPI (blue) in indicated cells. **B** Immunofluorescence staining showing the distribution of TFRC (green), EEA1 (red), RAB4 (red), RAB7 (red) and DAPI (blue) in indicated cells. **C-F** The distribution of EEA1 (red), RAB7 (red), TFRC (green) and DAPI (blue) in control and CCT3-knockdown cells. Histogram showing the statistical results for Pearson’s R correlation value from three biological replicates. **G** The expression of ACTN4 in indicted cells detected by Western blot were quantified and analyzed. **H-I** Western blot analysis of the knockdown efficiency of TFRC in 97L and LM3 cells. Histogram showing the statistical results from three biological replicates. **J** Cell viability analysis of control, CCT3-knockdown, and ACTN4-knockdown LM3 cells following treatment with Sorafenib for 24 h. **K-L** Indicated LM3 cells were treated with Sorafenib (14 µM) for 12 h, and then iron accumulation were measured. Histogram showing the statistical results from three biological replicates.


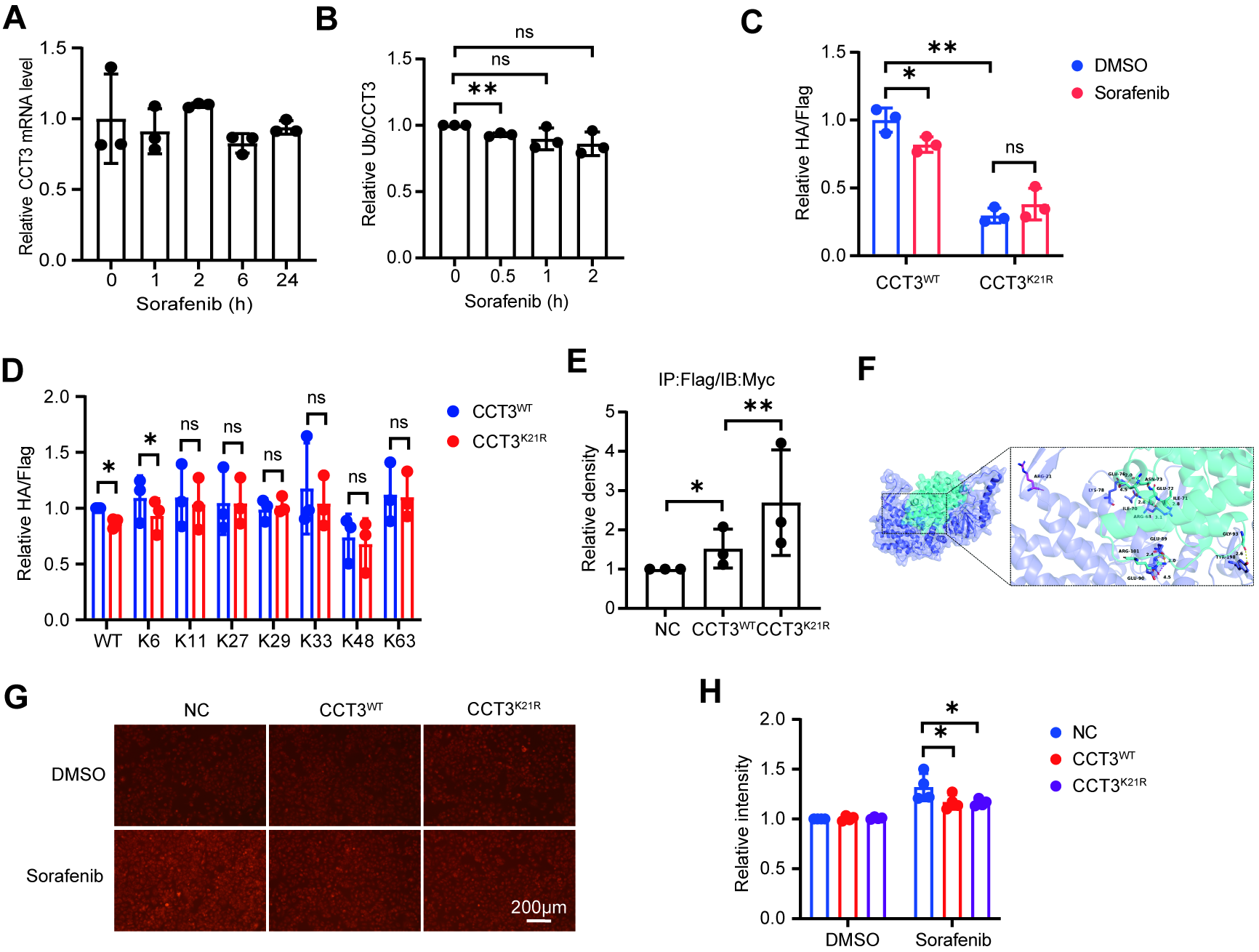


**Fig. S6 K21 ubiquitination was essential for CCT3. A** The mRNA levels of CCT3 after Sorafenib treatment was quantified and analyzed from three replicates in 97L cells**. B** The ubiquitinated CCT3 after Sorafenib treatment was quantified and analyzed from three biological replicates in 97L cells. **C** The ubiquitination of wild-type or K21R mutant CCT3 after Sorafenib treatment was quantified and analyzed from three biological replicates in 293T cells. **D** Ubiquitination levels of exogenous CCT3 measured by immunoprecipitation-western blot assay was quantified and analyzed from three biological replicates in 293T cells. **E** Quantification and statistics of ACTN4 interactions with wild-type or K21R mutant CCT3 in 293T cells. **F** Molecular docking of the CCT3^K21R^ (slate cartoon) and ACTN4(cyan cartoon) interaction complex, with corresponding-colored stick structures representing the binding sites. **G-H** LM3 cells overexpressed wild-type or K21R mutant CCT3 were treated with Sorafenib (14 µM) for 12 h, and then iron accumulation were measured. Histogram showing the statistical results from three biological replicates.
